# Loss of ABCA4 from photoreceptor discs is associated with glial transcriptomic changes in retinal organoids

**DOI:** 10.1093/stmcls/sxag045

**Published:** 2026-08-11

**Authors:** Rossella Valenzano, Andrew McDonald, Carmen Gallego, Charlotte A Andriessen, Ioannis Moustakas, Aat A Mulder, Harald M M Mikkers, Roman I Koning, Hailiang Mei, Jan Wijnholds

**Affiliations:** Department of Ophthalmology, Leiden University Medical Center, 2333 ZA Leiden, The Netherlands; Department of Ophthalmology, Leiden University Medical Center, 2333 ZA Leiden, The Netherlands; Department of Ophthalmology, Leiden University Medical Center, 2333 ZA Leiden, The Netherlands; Present address: VectorY Therapeutics, 1098 XH Amsterdam, The Netherlands; Department of Ophthalmology, Leiden University Medical Center, 2333 ZA Leiden, The Netherlands; Department of Biomedical Data Sciences, Sequencing Analysis Support Core, Leiden University Medical Center, 2333 ZA Leiden, The Netherlands; Department of Cell and Chemical Biology, Electron Microscopy Facility, Leiden University Medical Center, 2333 ZA Leiden, The Netherlands; Department of Cell and Chemical Biology, Leiden University Medical Center, 2333 ZA Leiden, The Netherlands; Department of Cell and Chemical Biology, Electron Microscopy Facility, Leiden University Medical Center, 2333 ZA Leiden, The Netherlands; Department of Biomedical Data Sciences, Sequencing Analysis Support Core, Leiden University Medical Center, 2333 ZA Leiden, The Netherlands; Department of Ophthalmology, Leiden University Medical Center, 2333 ZA Leiden, The Netherlands

**Keywords:** pluripotent stem cells, targeted gene disruption, differentiation, glia, retinal photoreceptors

## Abstract

**Background:**

Loss-of-function mutations in the *ABCA4* gene cause Stargardt disease (STGD1), the most common inherited macular dystrophy leading to progressive central vision loss.

**Methods:**

Here, we generated human induced pluripotent stem cell–derived retinal organoids harboring a premature stop codon in exon-24 of *ABCA4* to evaluate the impact of this mutation on mRNA and protein levels in a human model.

**Results:**

Immunofluorescence analysis revealed the absence of ABCA4 protein in the mutant photoreceptor outer segment discs, while single-cell RNA sequencing detected no major transcriptional alterations in rods and cones. Unexpectedly, differential gene expression and pathway enrichment analyses of Müller glial cells and astrocytes highlighted disruption of neuronal development, microenvironment of glial cells, intercellular communication, and programmed cell death pathways.

**Conclusions:**

These findings suggest that ABCA4 deficiency in photoreceptor discs may trigger early stress-associated transcriptomic responses in retinal glial cells prior to overt photoreceptor degeneration, potentially contributing to Stargardt disease pathogenesis.

## Graphical abstract

**Graphical Abstract:**
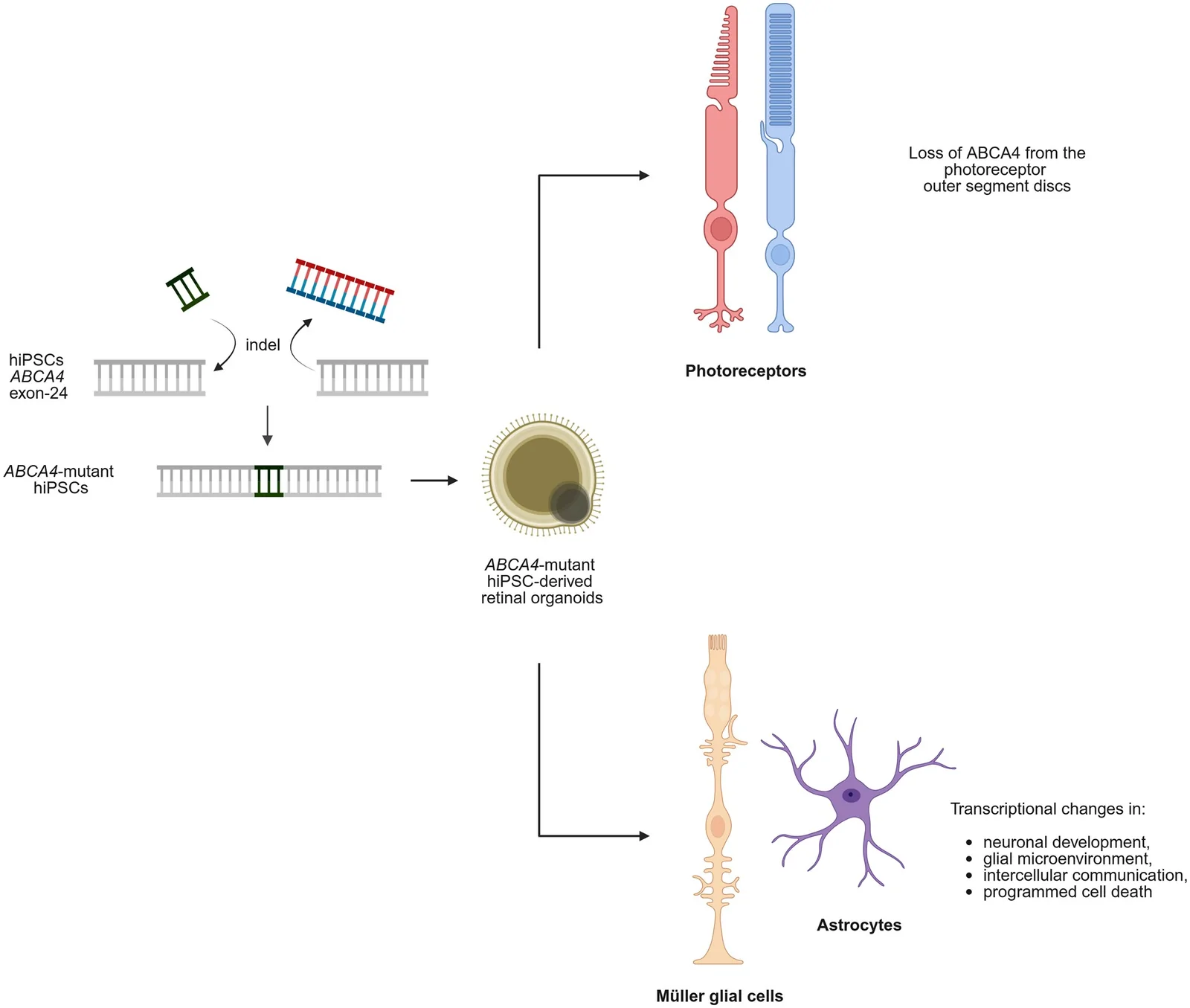

Significance statement Human induced pluripotent stem cell (hiPSC)–derived retinal organoids provide a powerful platform to investigate inherited retinal diseases. In this study, we generated *ABCA4*-mutant hiPSC lines and differentiated them into retinal organoids to model Stargardt disease. Despite loss of ABCA4 protein from the photoreceptor outer segment disks, rods and cones exhibited minimal transcriptional alterations. In contrast, the *ABCA4* variant was found to be associated with transcriptional changes in the glial cell homeostasis, suggesting that Müller glial cells and astrocytes might exhibit an early response to photoreceptor dysfunction in the absence of ABCA4.

## Introduction

The ATP-binding cassette, sub-family A, member 4 protein, also known as ABCA4, plays a critical role in the recycling of metabolites in the visual cycle. ABCA4 is predominantly localized to the membranous disks of photoreceptor outer segments, where it acts as a flippase, transporting N-retinylidene phosphatidylethanolamine (N-Ret-PE) from the luminal to the cytoplasmic leaflet of disk membranes, allowing the clearance of potentially toxic compounds.^1–3^ Loss-of-function of ABCA4 protein leads to the accumulation of N-Ret-PE in the outer segment disks, where it is converted to phosphatidyl-pyridinium bisretinoid (A2PE). Following disk shedding and phagocytosis by the retinal pigment epithelium (RPE), A2PE is hydrolyzed to the toxic bisretinoid A2E,^4^^,^^5^ which contributes to RPE dysfunction and subsequent photoreceptor degeneration.^6^^,^^7^

The *ABCA4* gene comprises 50 exons encoding a 2273 aa transmembrane protein.^8^^,^^9^ Variants are commonly associated with Stargardt disease (STGD1), an autosomal recessive retinal dystrophy causing vision loss in children and young adults, with a reported prevalence of 1 in 8000-10000.^10^ More than 1200 pathogenic *ABCA4* variants have been reported, resulting in a broad spectrum of phenotypes.^5^^,^^11^ Less frequently, *ABCA4* mutations are also implicated in other retinal disorders.^12^

Currently, there is no approved cure for STGD1. However, numerous clinical trials are ongoing, investigating diverse therapeutic strategies. Gene-based approaches include adeno-associated viral (AAV)-mediated therapies such as ACDN-01, which delivers an *ABCA4* RNA exon editor,^13^ SB-007, expressing functional *ABCA4*,^14^ and HG005, employing a dual-AAV system to restore full-length *ABCA4* expression.^15^ In addition, SAR422459 utilizes a lentiviral vector for *ABCA4* delivery and is undergoing long-term follow-up evaluation.^16^ Other therapies aim to reduce toxic retinoid accumulation, including Tinlarebant (LBS-008), a retinol-binding protein 4 antagonist currently in a phase 3 trial,^17^ and ALK-001, a deuterated vitamin A derivative designed to slow the formation of harmful vitamin A dimers.^18^ Broader approaches include OCU410ST, an AAV5-based gene therapy expressing human *RORA* to modulate lipid metabolism, oxidative stress, and inflammation,^19^ and metformin, which is being investigated for its potential to improve lysosomal function and fatty acid oxidation in the RPE.^20^

The high genotypic and phenotypic variability of *ABCA4*-related retinal dystrophies has posed a challenge for accurate disease modeling. Mice models, particularly *Abca4* knockout and disease-specific knock-in strains, are the most widely used in vivo models for STGD1.^21–25^ They have been instrumental in elucidating the ABCA4 transporter function, the bisretinoid accumulation, and the lipofuscin formation within the RPE. They have also served as key platforms to test therapeutic strategies.^21^^,^^23^^,^^26^ However, mice lack a macula and possess a rod-dominant retina, limiting their ability to fully model the macular degeneration and cone dysfunction that characterize human STGD1. In addition, the retinal phenotype in mice is generally milder than observed in patients, with a late onset that makes this model more suitable to study age-related macular degeneration (AMD).^27^ To overcome these limitations, additional models have been developed. Although rats also lack a true macula, their retinal organization and larger eye size facilitate surgical manipulation and imaging studies.^28^^,^^29^ Zebrafish provide a cone-rich retina, which more closely mirrors the human retina composition,^30–32^ while dogs possess the cone-enriched area centralis, analogous to the human macula.^33^ Pig models have also been used to test retinal gene therapy with dual-AAV intein vectors,^34^ achieving better results over other dual AAV-based strategies.^35^

Advances in stem cell technology have enabled the generation of human induced pluripotent stem cell (hiPSC)–derived retinal organoids that resemble human retinal architecture and contain all major retinal cell types.^36^^,^^37^ These organoids have been used to model inherited retinal dystrophies and investigate disease mechanisms.^38–41^ Importantly, the presence of rudimentary photoreceptor outer-segments and disk-like structures makes them a valuable tool for studying STGD1.^42^^,^^43^ Paired with CRISPR/Cas9 technology, retinal organoids allow the generation of precise loss-of-function models and offer a platform to evaluate gene augmentation and gene editing therapies in a human-derived system.^40^^,^^44^^,^^45^

In this study, we used CRISPR/Cas9 to introduce a premature stop codon in exon-24 of *ABCA4* in human iPSCs. Exon-24 was strategically selected due to its location in the middle of the *ABCA4* coding sequence, enabling the potential development of independent N- and C-terminal rescue strategies from a single mutation site via homology-independent targeted integration (HITI).^46^ The resulting *ABCA4* hiPSC-derived retinal organoids therefore constitute a valuable disease model for future testing of HITI-based CRISPR *ABCA4* gene editing strategies. The mutant organoids showed loss of ABCA4 from the photoreceptor disks. Single-cell RNA sequencing data revealed an unexpected response of Müller glial cells (MGCs) and astrocytes to the mutation, with transcriptional changes suggestive of altered regulation of apoptosis, cell-to-cell communication, and neuronal development pathways. These data provide new insights into the potential role of glial cells as therapeutic targets for the treatment of Stargardt disease.

## Methods

### hiPSC genome editing by CRISPR/Cas9

The gRNA targeting exon-24 of *ABCA4* was selected using the integrated DNA technology (IDT) Custom Alt-R™ CRISPR-Cas9 guide RNA online tool (https://eu.idtdna.com/). A single-stranded oligodeoxynucleotide (ssODN) HDR template (IDT) was designed to introduce an in-frame premature stop codon followed by a 10 bp deletion. Genome editing was performed on the parental LUMC0004iCTRL10 hiPSCs^47^ as previously described.^48^ Additional information can be found in Tables S1-S3.

### Cell culture and retinal organoid differentiation

hiPSCs were cultured on Matrigel-coated 6-well plates in mTeSR plus and incubated at 37 °C and 5% CO_2._ Cells were passaged following incubation with Gentle Cell Dissociation reagent (GCDR) (STEMCELL Technologies) and mechanical scraping. Retinal organoid differentiation followed an established protocol.^48^ Detailed information is provided in the Supplementary Methods and Table S3.

### Immunofluorescence analysis

Organoids were fixed with 4% paraformaldehyde in phosphate buffered saline (PBS) for 20 minutes followed by a brief washing in PBS. Fixed organoids were cryopreserved in 15% sucrose PBS solution for 30 minutes at RT, followed by incubation in a 30% sucrose PBS solution for at least 1 h at RT. The organoids were then frozen in Tissue-Tek O.C.T. Compound (Sakura Finetek Europe) and stored at −20°C. Cryosections were cut at 8 µm thickness using a Leica CM1900 cryostat (Leica Microsystems). Immunofluorescence analysis was performed as previously described.^49^ Sections were imaged on a Leica TCS SP8 confocal microscope (Leica Microsystems) using the Leica Application suite X (v3.7.0.20979). Antibodies used in this study are listed in Table S4.

### Quantification and statistical analysis

For quantification purposes, 3 representative images per organoid were acquired at 40× magnification. Quantification was performed using Fiji ImageJ (v2.17). Graphs were generated on GraphPad Prism (v10.2.3), where each data point corresponds to the mean per organoid ± standard error of the mean. The number of organoids tested is indicated in the figure legends. Experiments were performed on 3 independent rounds of differentiation, unless stated otherwise. Group differences were assessed using the Kruskal–Wallis test followed by Dunn’s multiple comparisons test. Significance was indicated as *P *< .05 (*), *P *< .01 (**), *P *< .001 (***), and ns (not significant).

### Single-cell RNA sequencing

Single-cell RNA sequencing was performed using the 10X Genomics Chromium 3′ gene expression platform. Organoids from a single differentiation batch were analyzed (ISO-CTRL *n* = 4; *ABCA4*-H10 *n *= 5; *ABCA4*-G3 *n *= 5). Individual organoids were dissociated into single-cell suspensions using the Papain Dissociation kit (Worthington Biochemical Corp.) following manufacturer’s instructions, and processed with Chromium v3 chemistry. Sequencing data were processed using Cell Ranger (v7.1.0) with the GRCh38 human reference genome. Downstream analyses were conducted in R (v4.4.0) using a Seurat-based pipeline. Differential gene expression was evaluated at the single-cell level using the Wilcoxon rank-sum test, with multiple-testing correction performed using the Benjamini–Hochberg method. Genes with an adjusted *P*-value < .05 were considered significantly differentially expressed. Additional analytical details are provided in the Supplementary Methods.

## Results

### Generation of CRISPR/Cas9-engineered hiPSCs harboring a premature stop codon in exon-24 of *ABCA4*

**Figure 1.**
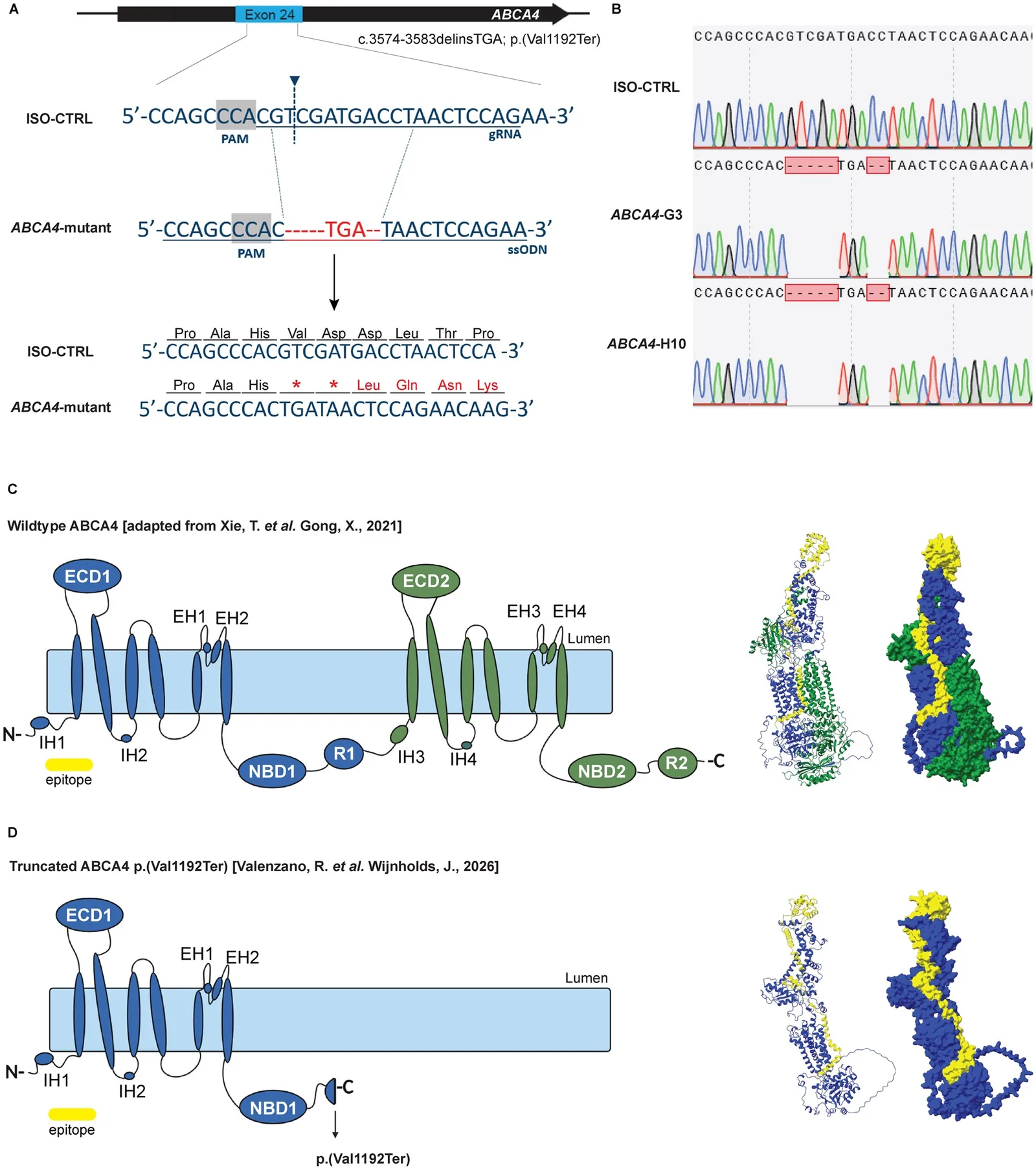
Generation of *ABCA4* hiPSCs and protein structure prediction. (A) Schematics indicating the gene disruption design, with a gRNA targeting exon-24 of *ABCA4,* and a single-stranded oligodeoxynucleotide (ssODN) donor inducing the deletion of 10 bp and the insertion of the premature stop codon TGA. (B) Sanger sequencing of the region of interest in exon-24 confirming the introduction of the mutation in homozygous form in *ABCA4* hiPSCs. (C, D) Depiction of the predicted protein domains in wildtype ABCA4 (adapted from Xie, T., Zhang, Z., Fang, Q. *et al*. Structural basis of substrate recognition and translocation by human ABCA4. *Nat Commun* 12, 3853 (2021) under the Creative Commons Attribution 4.0 International License, http://creativecommons.org/licenses/by/4.0/) (C) and truncated ABCA4 p.(Val1192Ter) (D). Left panels: Elongated ovals indicate transmembrane domains. NBD1 domain: 915-1152 aa; R1 domain: 1155-1279 aa. Right panels: predicted structures of the wild-type (C) and truncated protein (D) generated using AlphaFold and visualized in UCSF ChimeraX. Cartoon representations are color-coded to indicate protein regions: residues 250-1191 (blue), residues 1192-2273 (green). The ABCA4 antibody recognizes an epitope within residues 1-249 (yellow). ECD, extracellular domain; EH, extracellular helix; IH, intracellular helix; NBD, nucleotide-binding domain; R, regulatory domain.

Here, we applied the CRISPR/Cas9 gene editing tool to a previously characterized parental cell line, LUMC0004iCTRL10,^47^ renamed as “ISO-CTRL,” to generate homozygous mutant hiPSC lines where a 10-bp deletion-insertion results in the introduction of an in-frame premature stop codon in *ABCA4* exon-24, c.3574-3583delinsTGA; p.(Val1192Ter) (Figure 1A). Two independent mutant hiPSC subclones, labeled as *ABCA4*-G3 and *ABCA4*-H10, were analyzed by Sanger sequencing to confirm the presence of the mutation (Figure 1B). Based on the structural insights into ABCA4,^50^ this variant is predicted to generate a truncated ABCA4 protein within the regulatory domain 1 (R1; amino acids 1155-1279), located downstream of the nucleotide-binding domain 1 (NBD1; amino acids 915-1152 aa) (Figure 1C and D). The antibody epitope lies within the retained N-terminal region of the truncated protein (1-249 aa, highlighted in yellow) and appears structurally accessible in the Alpha-Fold predicted model (Figure 1C and D). G-banding indicated normal karyotypes (Figure S1A), and genome copy number variation analysis did not detect frequently occurring chromosomal abnormalities (Figure S1B), suggesting absence of genetic aberrations in the *ABCA4*-G3 and *ABCA4*-H10 lines. Lastly, the short tandem repeats analysis revealed that the mutant clones indeed share their derivation from the isogenic control line (Figure S1C). Both *ABCA4*-G3 and *ABCA4*-H10 retained expression of the canonical pluripotency markers Oct3/4 (89.8%-92.5%), Nanog (84.0%-92.7%), and SSEA-4 (99.9%-100%), indicating preservation of the undifferentiated pluripotent state following gene editing (Figure S2A). Further characterization included the assessment of trilineage differentiation potential through directed differentiation into mesoderm (Brachyury, CDX2, Vimentin), endoderm (EOMES, FOXA2, GATA4), and ectoderm (Nestin, PAX6) (Figure S2B). To rule out the occurrence of off-target mutagenesis, we used the IDT software “CRISPR-Cas9 guide RNA design checker” prediction tool (https://eu.idtdna.com/) to identify the top 5 candidate off-target sites and confirmed the absence of indels in the genomic regions of the *ABCA4* subclones by Sanger sequencing (Figure S2C).

### ABCA4 hiPSC-derived retinal organoids show correct development of photoreceptor cells, but loss of ABCA4 protein in OS-like structures

**Figure 2.**
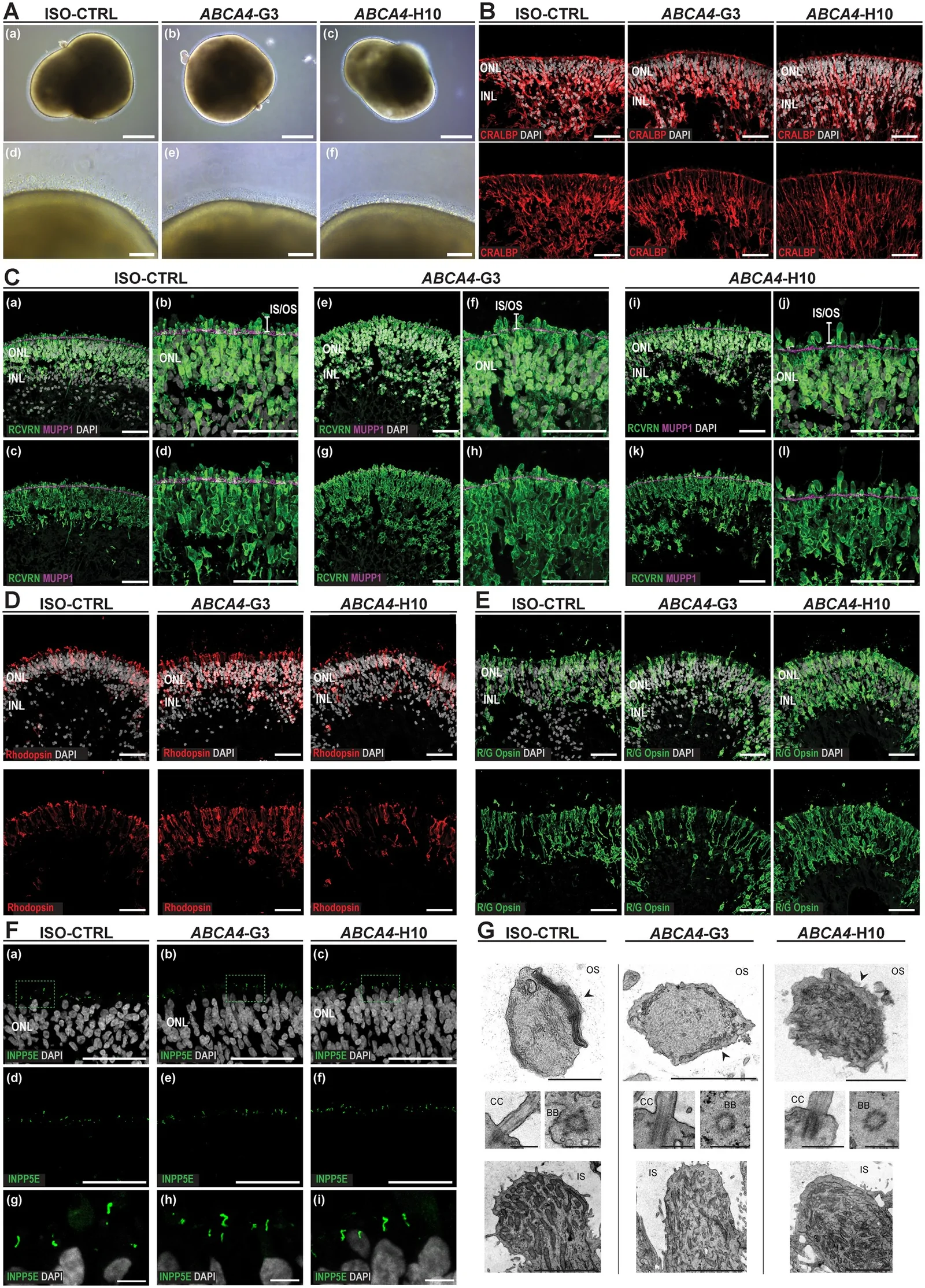
*ABCA4* hiPSC-derived retinal organoids show correct development of major retinal cell types at DD240. (A) Representative brightfield images of ISO-CTRL, *ABCA4*-G3 and *ABCA4*-H10 retinal organoids at DD240. Bottom images display the typical photoreceptor brush border at higher magnification. Scale bars in A(a-c) are 500 µm; scale bars in A(d-f) are 100 µm. (B) Representative immunofluorescence Z-stack images of CRALBP (red) with and without DAPI (grey). Scale bars: 20 µm. (C) Representative immunofluorescence Z-stack images of recoverin (green) and MUPP1 (purple), with and without DAPI (grey). Photoreceptor inner segment (IS) photoreceptor outer segment (OS). Scale bars in C(a, c, e, g, i, k) are 20 µm; scale bars in C(b, d, f, h, j, l) are 50 µm. (D) Representative immunofluorescence Z-stack images of rhodopsin (red), with and without DAPI (grey). Scale bars: 20 µm. (E) Representative immunofluorescence Z-stack images of red/green (R/G) opsin (green), with and without DAPI (grey). Scale bars: 20 µm. (F) Representative immunofluorescence Z-stack images of INPP5E (green), with and without DAPI (grey). Dashed boxes highlight regions shown at higher magnification in the bottom panel. Scale bars in F(a-f) are 50 µm; scale bars in F(g-i) are 5 µm. (G) Representative transmission electron microscopy (TEM) images of photoreceptors in ISO-CTRL and *ABCA4* retinal organoids at DD240. Upper panel: OS-like structures with arrows pointing to the disk membranes. Scale bars from left to right: 1 µm, 2 µm, 1 µm. Middle panel: connecting cilium (CC) and basal body (BB). Scale bars: 0.5 µm. Bottom panel: inner segment-like structures. Scale bars: 3 µm. INL, inner nuclear layer; ONL, outer nuclear layer.

After validation of the hiPSC subclones, the *ABCA4*-G3 and *ABCA4*-H10 cell lines were differentiated into retinal organoids, along with their isogenic parental line, until differentiation day (DD) 240, following an earlier established protocol.^51^ The brightfield images showed a comparable development of the retinal lamination in both isogenic control and mutant organoids, with the formation of a characteristic brush border corresponding to the photoreceptor layer (Figure 2A). We did not observe morphological changes in the *ABCA4*-G3 and *ABCA4*-H10 retinal organoids that would be indicative of retinal degeneration.

We previously demonstrated that organoids cultured using the same differentiation protocol as used in this study show correct development of all major retinal populations.^48^ To confirm the appropriate differentiation of the *ABCA4* mutant hiPSC lines into retinal organoids, we performed immunofluorescence analysis using antibodies against key markers of selected cell types. CRALBP staining highlighted the presence of MGCs, displaying the characteristic elongated, radial morphology, with filamentous processes extending from the inner retina toward their apical end feet (Figure 2B). Anti-recoverin marked the photoreceptor cells, with multiple PDZ domain protein 1 (MUPP1) antibody delineating the outer limiting membrane (OLM), allowing clear visualization of the inner (IS) and outer segments (OS) extending beyond it (Figure 2C). Ultimately, rods and cones were separately labeled by using antibodies against rhodopsin and R/G opsin, respectively, which are normally trafficked from the IS to the OS (Figure 2D and E). These 2 portions of the photoreceptor cells are known to be linked by the connecting cilium (CC), here detected in both control and *ABCA4* organoids via the inositol polyphosphate-5-phosphatase E (INPP5E) marker (Figure 2F). A deeper focus on the photoreceptor structure was provided by the transmission electron microscopy (TEM) analysis, performed according to an established protocol.^52^ The images showed the presence of the photoreceptor inner segment-like regions containing mitochondria, the CC and related basal body (BB), and very immature outer segment (OS)-like structures, characterized by the presence of yet poorly organized photoreceptor disks (Figure 2G). Overall, qualitative comparison did not reveal overt differences, suggesting that *ABCA4* mutation does not affect retinal organoid development.

**Figure 3.**
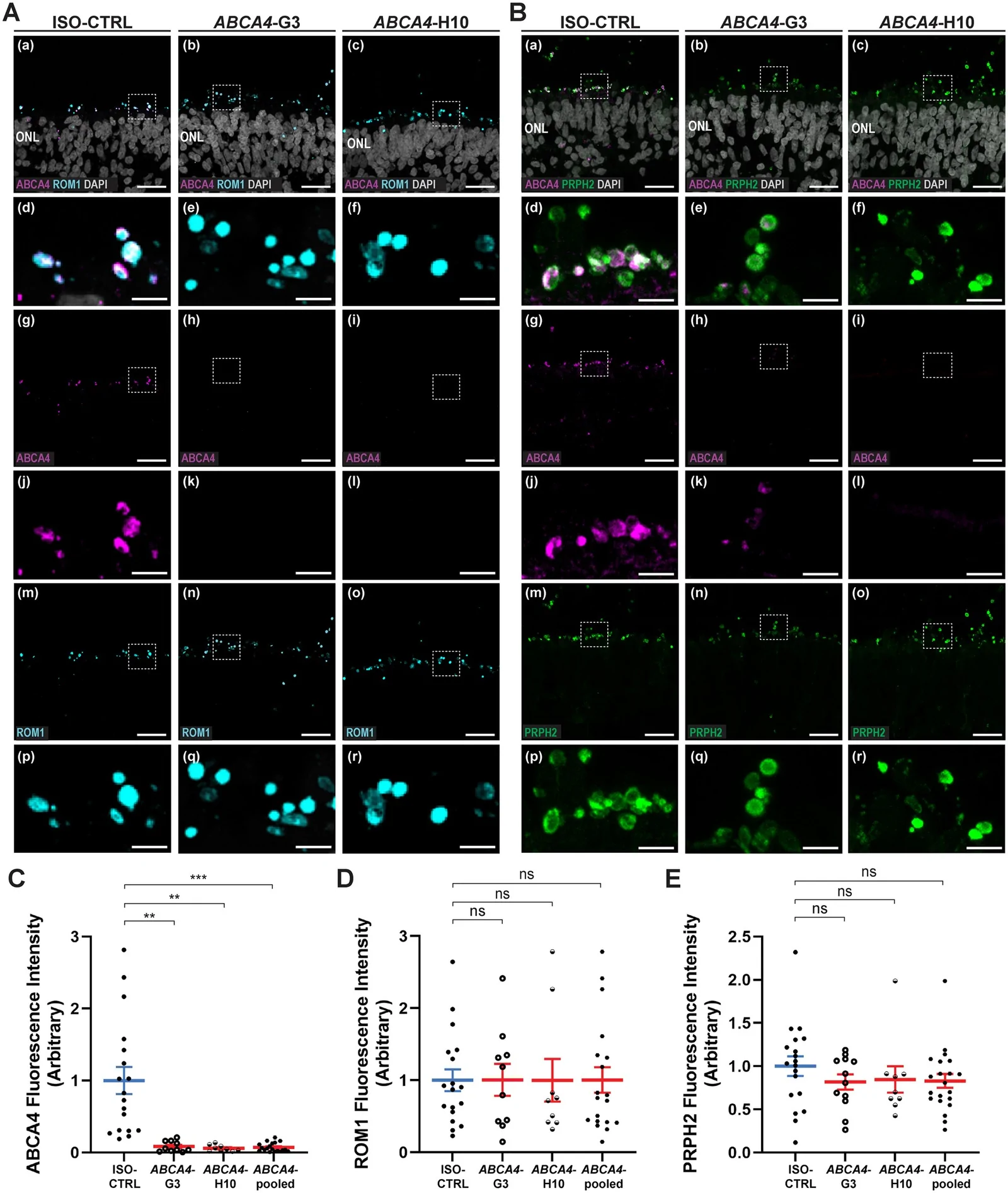
Loss of ABCA4 from the photoreceptor disks does not affect the localization of other markers in *ABCA4* retinal organoids at DD240. (A) Representative immunofluorescence images of ABCA4 (magenta) and ROM1 (cyan), with and without DAPI (grey), in ISO-CTRL, *ABCA4*-G3, and *ABCA4*-H10 retinal organoids at DD240. Regions outlined by dashed boxes are displayed at higher magnification in the corresponding zoomed panels below. Scale bars in A(a-c), A(g-i), A(m-o) are 20 µm; scale bars in A(d-f), A(j-l), A(p-r) are 5 µm. (B) Representative immunofluorescence images of ABCA4 (magenta) and PRPH2 (green), with and without DAPI (grey), in ISO-CTRL, *ABCA4*-G3, and *ABCA4*-H10 retinal organoids at DD240. Regions outlined by dashed boxes are displayed at higher magnification in the corresponding zoomed panels below. Scale bars in B(a-c), B(g-i), B(m-o) are 20 µm; scale bars in B(d-f), B(j-l), B(p-r) are 5 µm. (C) Quantitative analysis of ABCA4 intensity in ISO-CTRL and *ABCA4* retinal organoids. Number of organoids used: ISO-CTRL *n *= 18; *ABCA4*-H10 *n *= 9; *ABCA4*-G3 *n *= 10, from three rounds of differentiation. Group differences were assessed using the Kruskal-Wallis test followed by Dunn’s multiple comparisons test. Significance was indicated as *P *< .01 (**), and *P *< 0.001 (***). Statistical analysis: *P *= .0097, *P *= .0010, *P *= .0003, from left to right. (D) Quantitative analysis of ROM1 intensity in ISO-CTRL and *ABCA4* retinal organoids. Number of organoids used: ISO-CTRL *n *= 18; *ABCA4*-H10 *n *= 9; *ABCA4*-G3 *n *= 10, from three rounds of differentiation. Group differences were assessed using the Kruskal-Wallis test followed by Dunn’s multiple comparisons test. Significance was indicated as ns (not significant). Statistical analysis: *P *> .9999 for all groups. (E) Quantitative analysis of PRPH2 intensity in ISO-CTRL and *ABCA4* retinal organoids. Number of organoids used: ISO-CTRL *n *= 19; *ABCA4*-H10 *n *= 9; *ABCA4*-G3 *n *= 12, from three rounds of differentiation. Group differences were assessed using the Kruskal–Wallis test followed by Dunn’s multiple comparisons test. Significance was indicated as ns (not significant). Statistical analysis: *P *= .9231, *P *= .3875, *P *= .3932, from left to right. (C, D, E) All organoid measurements are displayed as individual datapoints for transparency and were treated as independent replicates.

Given the expected localization of ABCA4 to photoreceptor disks, we further characterized the outer segments using two disk markers, the rod outer segment membrane protein 1 (ROM1) and peripherin-2 (PHRP2). Interestingly, while at DD240 ABCA4 mostly co-localized with ROM1 (Figure 3A) and PRPH2 (Figure 3B) in the isogenic control organoids, the mutant photoreceptors of *ABCA4*-G3 and *ABCA4*-H10 displayed loss of ABCA4 signal at the outer segments. The anti-ABCA4 raised against the amino-terminal half of ABCA4 (amino acids 1-249) did not detect the truncated protein, suggesting that, if produced, this shorter ABCA4 may be degraded and/or mislocalized within the photoreceptor cells. Quantitative analysis across 3 independent differentiations confirmed a significant decrease of ABCA4 signal in mutant organoids (Figure 3C), while ROM1 (Figure 3D) and PRPH2 (Figure 3E) intensity levels remained unchanged. These results further support that ABCA4 deficiency does not impair the expression of key photoreceptor outer segments markers in retinal organoids.

To obtain complementary data on ABCA4 protein expression, we performed Western blot analysis on protein lysates obtained from control and mutant organoids. Efficient protein transfer onto the polyvinylidene fluoride membrane was confirmed by Ponceau S staining (Figure S3A). However, although previous studies have reported successful detection of ABCA4 protein in wild-type retinal organoids by Western blot using the same antibody as employed in this study,^53^ we were unable to reproduce these findings under our experimental conditions. Despite testing multiple protein inputs from individual and pooled organoid samples, and using both standard and high-sensitivity detection substrates, neither full-length nor truncated ABCA4 protein could be reliably detected in lysates from the control or *ABCA4*-variant retinal organoids generated with our differentiation protocol (Figure S3B), whereas the glyceraldehyde 3-phosphate dehydrogenase (GAPDH) was robustly detected in all samples (Figure SC).

### Retention of *ABCA4* transcripts in *ABCA4* retinal organoids

**Figure 4.**
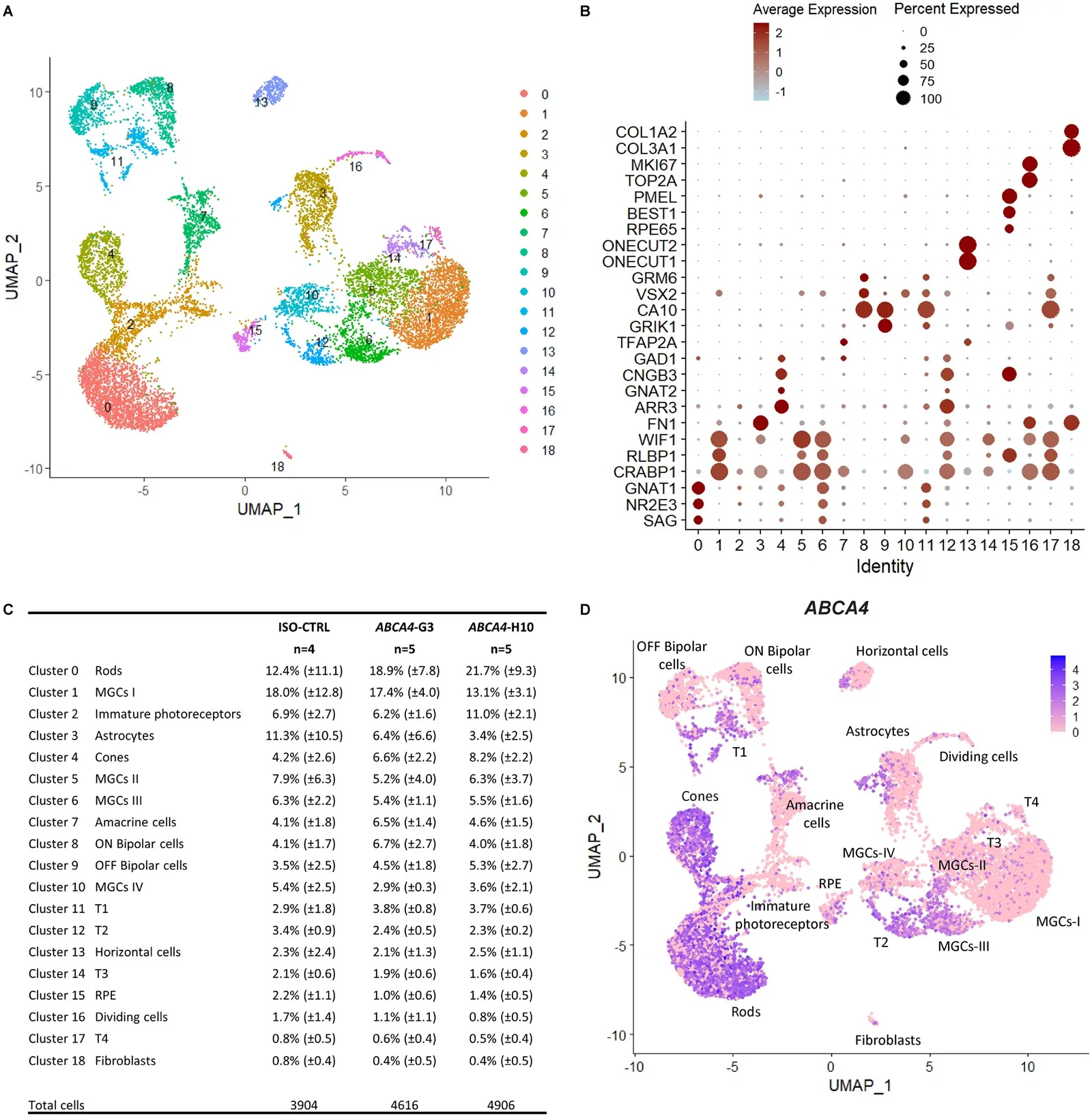
Cell-type identification and *ABCA4* transcript enrichment at DD225 in hiPSC-derived *ABCA4* and control retinal organoids by single-cell RNA sequencing. (A) UMAP displaying 19 distinct clusters from 14 scRNA-Seq experiments. (B) Dot plot showing transcript enrichment of cell-specific markers to assign cell identity. (C) Table indicating the percentage of each cell type across conditions including standard deviation. (D) UMAP plot showing *ABCA4* transcript enrichment. Number of organoids used: ISO-CTRL *n *= 4; *ABCA4*-H10 *n *= 5; *ABCA4*-G3 *n *= 5 from 14 scRNA-Seq of the same differentiation round.

To investigate potential transcriptomic alterations not associated with morphological defects, we performed single-cell RNA sequencing (scRNA-Seq) on control and *ABCA4* retinal organoids at DD225 (ISO-CTRL *n* = 4, *ABCA*4-G3 *n* = 5, *ABCA4*-H10 *n* = 5). By performing 14 scRNA-Seq experiments, we identified 19 distinct clusters displayed on a UMAP plot (Figure 4A), including rod and cone photoreceptors, MGCs, ON and OFF bipolar cells, horizontal cells, and amacrine cells. Cluster identities were assigned based on established marker gene expression (Figure 4B).^54^^,^^55^ Unexpectedly, several additional cell types were identified compared to the clusters found in a previous analysis conducted on organoids differentiated using the same protocol.^48^ Such cells were accounted for as astrocytes, dividing cells, fibroblasts, and multiple transient cell populations. These transient clusters comprised bipolar–photoreceptor cells (T1), cone–bipolar–horizontal cells (T2), MGC–cone cells (T3), and bipolar–amacrine–horizontal cells (T4). Multiple MGC clusters were detected and annotated as MGCs-I–IV. Cell-type proportions were comparable across ISO-CTRL and *ABCA4* organoids, indicating no major differences in cellular composition, further suggesting that the *ABCA4* variant does not significantly impact retinal organoid development (Figure 4C). The only exception was represented by the transient cluster T2, which abundance was found statistically significantly reduced in the *ABCA4*-H10 group.

*ABCA4* transcripts were found to be predominantly enriched in photoreceptor cells and transient populations, with lower expression levels detected in a subset of MGCs, amacrine cells, horizontal cells, astrocytes, and RPE (Figure 4D). Although the presence of a premature stop codon was expected to activate the nonsense-mediated mRNA decay, no statistically significant reduction in *ABCA4* transcript levels was detected in most cell types of *ABCA4* organoids (Figure S4A, S4D, S4G-I). The sole exception was the rod photoreceptors from the *ABCA4*-H10 subclone, where *ABCA4* was identified as a differentially expressed gene (DEG) (Figure S4A). Consistent with the immunofluorescence data showing preserved localization of ROM1 and PRPH2 proteins, transcript levels of these disk markers did not change in both rod and cone photoreceptors of *ABCA4* organoids (Figure S4B, C, E, and F).

Differential expression analysis revealed minimal transcriptional changes in the photoreceptor populations. Rods displayed a limited number of DEGs, only 5 in *ABCA4*-G3 (Figure S5A) and 37 genes in *ABCA4*-H10 (Figure S5B). Among these, *B2M* and the long non-coding RNA *AL451062.1* were the only genes consistently dysregulated across both *ABCA4* subclones. On the other hand, cone photoreceptors showed no DEGs in *ABCA4*-G3 (Figure S5C) and only 3 DEGs in *ABCA4*-H10 (*NRL*, *CC2D2A*, and *B2M*) (Figure S5D). These findings indicate that, despite the loss of ABCA4 protein from the photoreceptor disks, rods and cones in *ABCA4* hiPSC-derived retinal organoids largely retain a stable transcriptomic profile.

### scRNA-Seq analysis reveals major transcriptional response in ABCA4 MGCs and astrocytes

**Figure 5.**
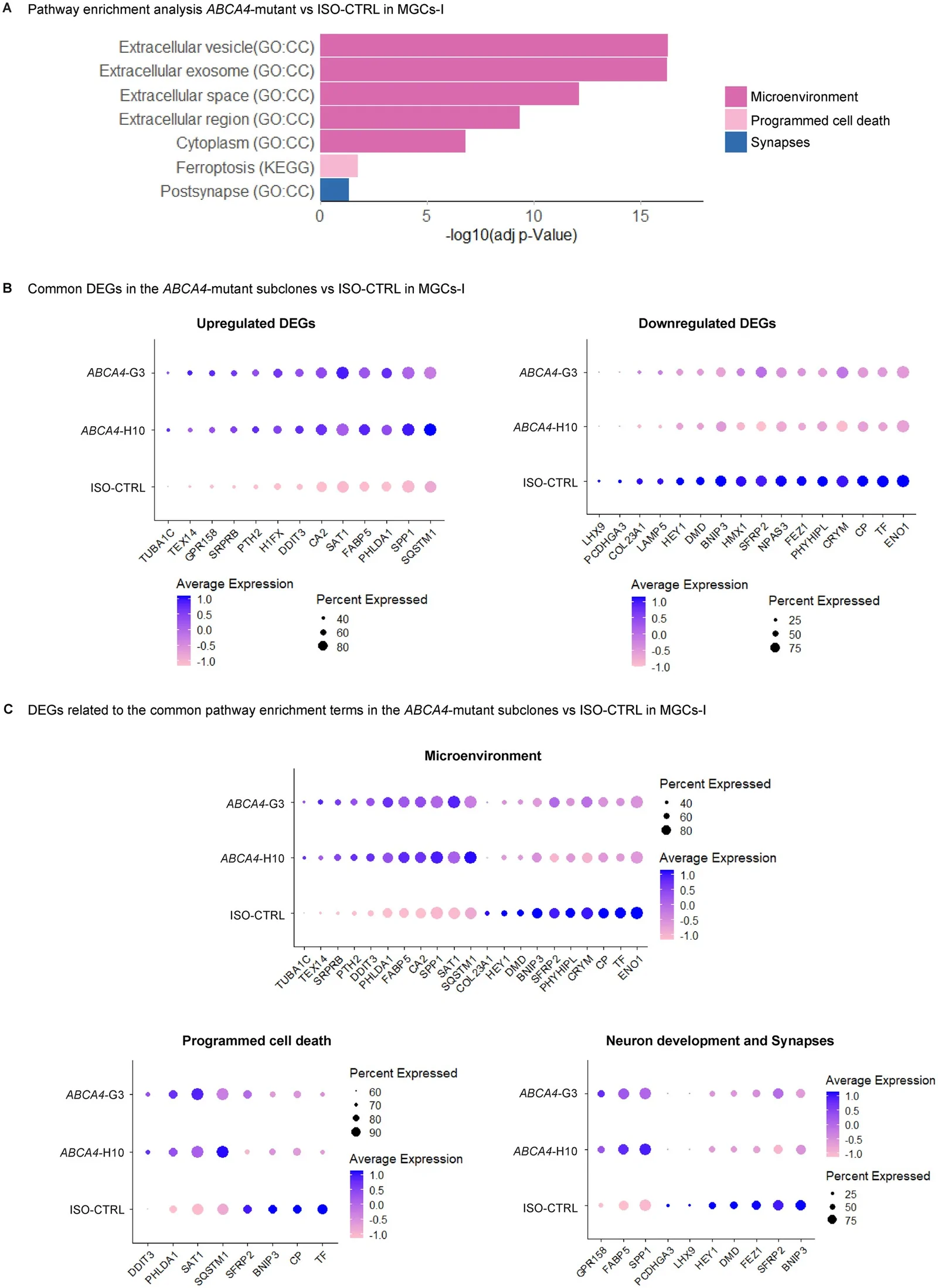
scRNA-Seq analysis shows a transcriptional response of Müller glial cells upon *ABCA4* mutation. (A) Pathway enrichment analysis of differentially expressed genes (DEGs) in MGCs-I, with similar terms grouped into three categories. (B) Dot plots of the statistically significant 13 upregulated DEGs (i) and 16 downregulated DEGs (ii) found in the MGCs of both *ABCA4* subclones. (C) Common DEGs involved in the shared pathway enrichment terms related to microenvironment (i), programmed cell death (ii), and neuron development and synapses (iii). Number of organoids used: ISO-CTRL *n *= 4; *ABCA4*-H10 *n *= 5; *ABCA4*-G3 *n *= 5 from 14 scRNA-Seq of the same differentiation round. Differential gene expression was evaluated at the single-cell level using the Wilcoxon rank-sum test, with multiple-testing correction performed using the Benjamini–Hochberg method. Genes with an adjusted *P*-value < .05 were considered significantly differentially expressed.

In contrast to photoreceptors, a substantial transcriptional response was observed in MGCs and astrocytes. In the MGCs-I cluster, *ABCA4*-G3 (Figure S6A) and *ABCA4*-H10 (Figure S6B) displayed 70 and 317 DEGs, respectively, with 29 genes commonly dysregulated across subclones. These included 13 upregulated genes, such as *SAT1*, *PHLDA1*, *FABP5*, and *SQSTM1* (Figure 5Ai), and 16 downregulated genes, including *TF*, *PCDHGA3*, *BNIP3*, and *HEY1* (Figure 5Aii). Similar expression patterns were observed across additional MGC clusters, with consistent downregulation of *TF* in MGCs-I, MGCs-II, and MGCs-III, and downregulation of *CRYM* in MGCs-I and MGCs-II. We also detected upregulation of glial markers such as *CD44* in MGCs-I and MGCs-III, and upregulation of *FTL* in MGCs-I, MGCs-II, MGCs-III, MGCs-IV, and T2. *IFITM3* was found upregulated in MGCs-II and MGCs-IV. In cluster MGCs-IV other interferon-induced genes as well were found upregulated, namely *IFITM1* and *IFITM2*.

*PCDHGA3,* encoding a small calcium-dependent neural cadherin-like cell adhesion protein, was markedly downregulated in all the *ABCA4* mutant 4 MGC clusters, along with astrocytes, amacrine cells, and the transient cluster T2, despite at low-level of expression. In some of these clusters, a complete loss of *PCDHGA3* expression was observed, suggesting impaired intercellular communication within the retinal network.

The DGEs identified in each cluster were subsequently used to perform pathway enrichment analyses comparing *ABCA4*-G3 vs ISO-CTRL and *ABCA4*-H10 vs ISO-CTRL. To increase the robustness of the analysis, only enriched terms present in both comparisons were included in the resulting bar plots.

Pathway enrichment analysis of MGCs-I revealed enrichment in terms related to “microenvironment,” such as extracellular vesicle and cytoplasm, “programmed cell death,” and particularly ferroptosis, and “synapses,” with an accent on the postsynaptic specialization (Figure 5B). We then investigated which of the common DEGs found in the MGCs-I cluster of *ABCA4*-G3 and *ABCA4*-H10 were associated with such categories and displayed them in dot plots (Figure 5C). For the microenvironment group, multiple DEGs were retrieved, including *FABP5*, *COL23A1*, *SFRP2*, and *CRYM* (Figure 5Ci), whereas enrichment of programmed cell death was driven by *PHLDA1*, *BNIP3* (Figure 5Cii). The post-synapse, considered one of the later stages of the broader process of neuronal development, was associated with DEGs such as *SPP1*, *LHX9*, and *FEZ1* (Figure 5Ciii). Notably, *PCDHGA3* was also linked to this category and was consistently downregulated.

**Figure 6.**
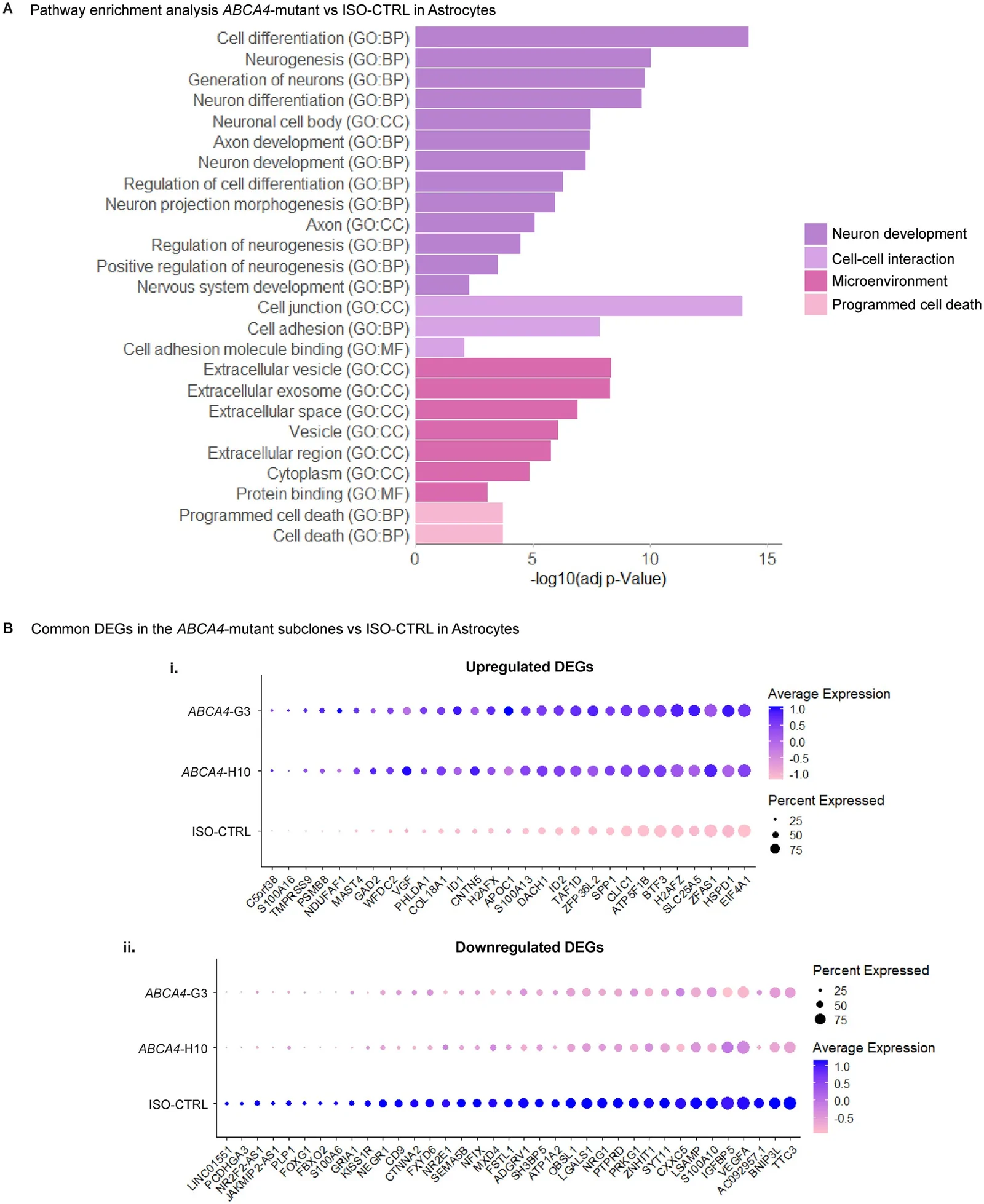
Astrocytes transcriptional profile is strongly modified upon *ABCA4* gene disruption. (A) Pathway enrichment analysis of differentially expressed genes (DEGs) in astrocytes, with similar terms grouped into four categories. (B) Dot plots of the statistically significant 29 upregulated DEGs (i) and 37 downregulated DEGs (ii) found in the astrocytes of both *ABCA4* subclones. Number of organoids used: ISO-CTRL *n *= 4; *ABCA4*-H10 *n *= 5; *ABCA4*-G3 *n *= 5 from 14 scRNA-Seq of the same differentiation round. Differential gene expression was evaluated at the single-cell level using the Wilcoxon rank-sum test, with multiple-testing correction performed using the Benjamini–Hochberg method. Genes with an adjusted *P*-value < .05 were considered significantly differentially expressed.

**Figure 7.**
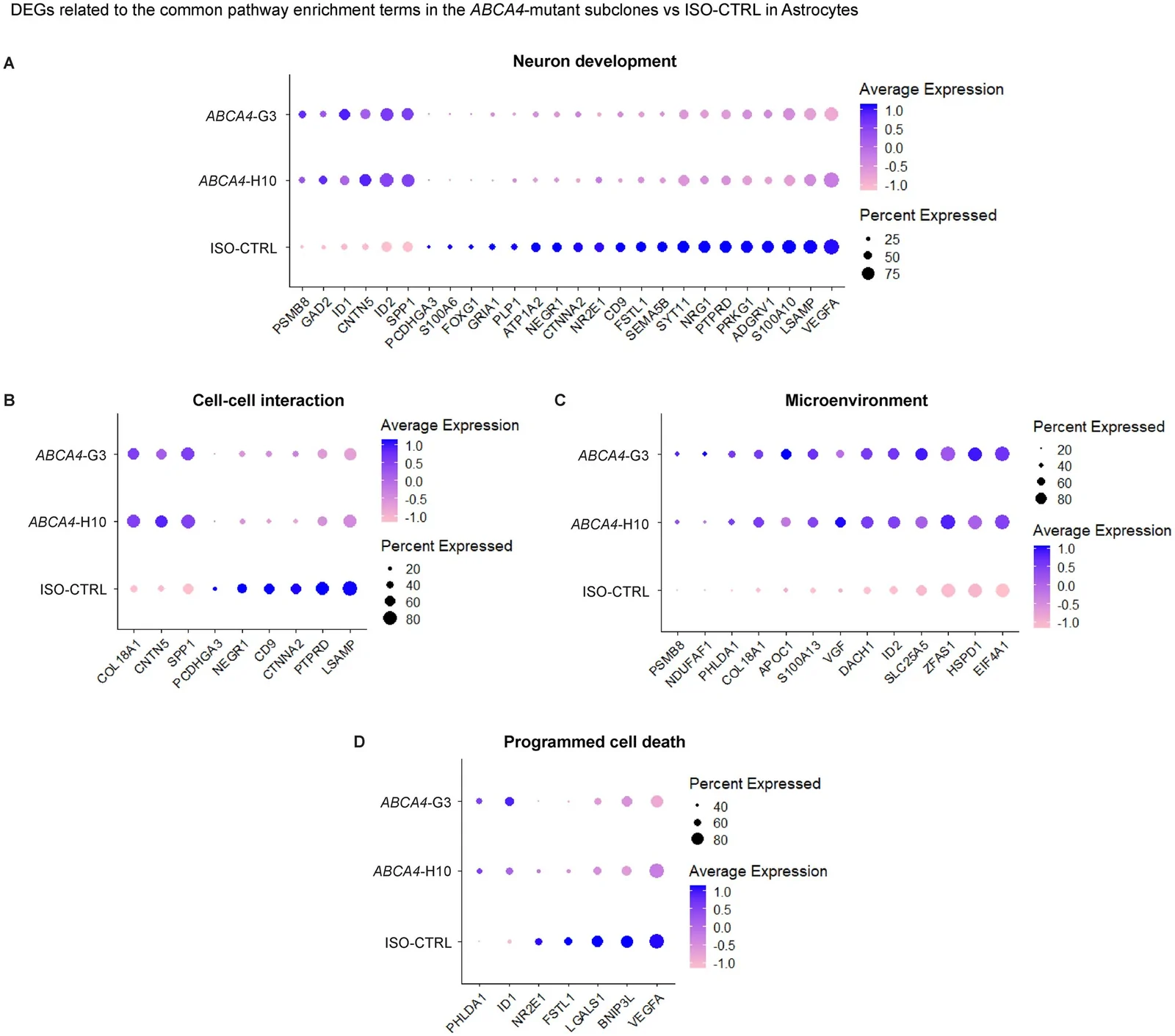
Differential expression analysis of *ABCA4* mutant astrocytes compared to isogenic controls. (A-D) Common DEGs involved in the shared pathway enrichment terms related to neuron development (A), cell-cell interaction (B), microenvironment (C), and programmed cell death (D). Number of organoids used: ISO-CTRL *n *= 4; *ABCA4*-H10 *n *= 5; *ABCA4*-G3 *n *= 5 from 14 scRNA-Seq of the same differentiation round. Differential gene expression was evaluated at the single-cell level using the Wilcoxon rank-sum test, with multiple-testing correction performed using the Benjamini–Hochberg method. Genes with an adjusted *P*-value < .05 were considered significantly differentially expressed.

Astrocytes exhibited the strongest transcriptional alteration, with 113 DEGs in *ABCA4*-G3 (Figure S6C) and 447 DEGs in *ABCA4*-H10 (Figure S6D), of which 66 genes were shared between subclones (Figure 6A). These common DEGs included *PHLDA1*, *ADGRV1*, *BNIP3L*, and *NR2E1,* functionally overlapping with those identified in the MGCs. The pathway enrichment analysis performed on the astrocytes cluster revealed enrichment in terms related to “neuron development,” “cell-cell interaction,” “microenvironment,” and “programmed cell death” (Figure 6B). Among the DEGs contributing to neuronal development pathways, *NEGR1*, *NR2E1*, *NRG1,* and *ADGRV1* were downregulated (Figure 7A). *PCDHGA3* was again found to be downregulated, implicating impaired cell-cell interactions (Figure 7B). Enrichment of microenvironment-related terms involved genes such as *COL18A1* and *APOC1* (Figure 7C), whereas *PHLDA1* and *BNIP3L* contributed to the dysregulation of programmed cell death (Figure 7D).

Given the well-known heterogeneity of organoids, it is important to be reminded that separate clones may manifest the phenotype differently, with some exhibiting a stronger response to the mutation, and others being more tolerant. In fact, pathway enrichment analyses for clones *ABCA4*-G3 and *ABCA4*-H10 also included uniquely enriched terms. In the MGCs-I cluster of *ABCA4*-H10 organoids, besides those terms grouped under the shared categories of “microenvironment,” “programmed cell death,” and “synapses,” we identified additional terms related to “cell-cell interaction,” more broad “neuron development,” “iron binding and transport,” and “cell redox homeostasis” (Figure S7A). Moreover, the “programmed cell death” category expanded to include pathways related not only to ferroptosis but also to apoptosis, another form of regulated cell death. On the other hand, *ABCA4*-G3 showed enrichment in pathways related to the “respiratory chain” and unique molecular function terms associated with “retinoid binding” (Figure S7B). DEGs underlying these subclone-specific pathways are shown in Figure S8. The enrichment of “cell redox homeostasis” and “respiratory chain” pathways in *ABCA4*-H10 and *ABCA4*-G3, respectively, suggests the presence of ongoing metabolic stress in both subclones.

We next assessed the potential presence of an overall effect on the MGCs-I at transcriptional level by comparing the pool of 10 *ABCA4*-mutant versus the 4 ISO-CTRL organoids. The differential expression gene analysis identified 192 DEGs for this cluster and the pathway enrichment analysis returned gene ontology (GO) terms belonging to the categories of “microenvironment,” “neuron development,” “cell-cell interaction,” “iron binding and transport,” and “programmed cell death” (Figure S9), confirming the disruption of pathways already displayed in the previous bar plots for MGCs. Representative DEGs are shown in Figure S10.

Similarly, as shown for the MGCs-I, we observed additional subclone-specific pathway enrichments in the astrocytes. Besides the shared categories of “neuron development,” “cell-cell interaction,” “microenvironment,” and “programmed cell death,” extra terms related to “synapses” and postsynaptic specialization were exclusively enriched in *ABCA4*-H10 organoids (Figure S11A), whereas *ABCA4*-G3 astrocytes displayed enrichment in “glycolysis” and, indirectly, in pathways linked to the “respiratory chain complex” (Figure S11B). Corresponding supplemental dot plots are provided in Figure S12.

When pooling all mutant organoids in comparison to the controls, we found 441 DEGs in the astrocytes cluster, associated with “neuron development,” “microenvironment,” “cell-cell interaction,” and “programmed cell death” (Figure S13). The GO terms found in the pooled analysis shared identity with the disrupted pathways already shown in Figure 6, with associated DEGs displayed in Figure S14.

Further studies are required to determine whether the transcriptional alterations observed in MGCs and astrocytes translate into changes at the protein level. As a preliminary exploratory analysis, we investigated the presence of gliosis in mutant organoids using glial fibrillary acidic protein (GFAP) together with CD44, a marker expressed in the end-feet processes of MGCs (Figure S15A). Although qualitative differences were observed, statistical analysis did not reveal significant alterations between control and mutant organoids (Figure S15B and C), likely due to the limited number of organoids and differentiation batches analyzed (*n* = 2).

Given that pathway enrichment analysis also highlighted disruptions in the programmed cell death, we next assessed apoptosis by quantifying cells positive for cleaved caspase-3. Punctate cleaved caspase-3 signals were detected throughout the organoids (Figure S15D), and positive cells were quantified across the outer nuclear layer (ONL), inner nuclear layer (INL), and inner retina in both control and mutant groups. However, the qualitative differences observed between conditions did not reach statistical significance (Figure S15E). More independent batches will be required to determine the existence of a consistent biological effect and validate the key findings at protein level for MGCs and astrocytes.

## Discussion

The aim of this study was to investigate the effects of *ABCA4* gene disruption on transcript and protein expression in hiPSC-derived retinal organoids. Although the introduction of a premature stop codon in exon-24 was predicted to generate a truncated ABCA4 protein within the regulatory domain 1, ABCA4 was undetectable in the photoreceptor outer segment disks when using an antibody targeting its N-terminal half. The ABCA4 loss was associated with altered transcriptional signatures in MGCs and astrocytes, particularly affecting pathways involved in neuronal development, glial microenvironment, and programmed cell death.

As part of the retinal organoid characterization workflow, immunofluorescence analyses were performed at DD240 to allow further photoreceptor maturation, whereas scRNA-Seq was conducted at DD225 to ensure comparability with our previously published datasets. As they represent closely related late-stage maturation time points, we do not expect substantial developmental differences that would affect the interpretation of the results.

Immunofluorescence analysis showed that homozygous *ABCA4* disruption did not alter the expression of major retinal cell markers compared with isogenic controls. Consistent with the absence of RPE and associated A2E toxicity, no morphological abnormalities were observed in *ABCA4*-G3 and *ABCA4*-H10 organoids.^4^^,^^7^^,^^56^ TEM confirmed preserved photoreceptor ultrastructure, including connecting cilia, basal bodies, and IS- and OS-like structures. Although ABCA4 protein was lost at the photoreceptor outer segment disks, the localization of the disk markers ROM1 and PRPH2 remained unaffected.

Unexpectedly, scRNA-Seq revealed that *ABCA4* transcript levels were not significantly reduced in mutant photoreceptors, with the exception of rods from the *ABCA4*-H10 subclone in which *ABCA4* was identified as a DEG. This suggests that the mutant transcripts may not undergo complete nonsense-mediated decay (NMD), allowing translation of a truncated protein no longer able to maintain its physiological localization. However, transcript abundance alone does not demonstrate NMD escape, and additional experiments involving pharmacological inhibition of NMD would be useful to directly assess the extent of transcript degradation and validate this mechanism. Because Western blot analysis was technically unsuccessful, we cannot exclude expression of a truncated, potentially misfolded ABCA4 protein below the detection limit of our immunofluorescence analysis. If present, it could engage in aberrant interactions with other retinal cellular components and interfere with biological functions. Consequently, the observed transcriptional alterations cannot be unequivocally attributed to the absence of functional ABCA4 alone. Throughout this study, “loss of ABCA4” therefore refers to the absence of detectable ABCA4 localization at the photoreceptor outer segments, rather than definitive absence of any forms of ABCA4.

Overall, mutant rods and cones did not exhibit major transcriptional alterations. One of the few dysregulated genes in the rod photoreceptors was *B2M*, encoding a component of MHC class I molecules typically associated with immune signaling. Its upregulation in photoreceptor cells may reflect an intrinsic cellular stress response as a hallmark of neuronal stress. However, the mechanistic significance of this observation remains to be unveiled.

Interestingly, low-level *ABCA4* expression was also detected in several non-photoreceptor retinal cell types at DD225, including MGCs, amacrine cells, horizontal cells, and astrocytes, consistent with previous reports from retinal organoids cultured for up to 300 days,^57^ as well as with single-cell transcriptomic datasets from both fetal and adult human retina.^58^ Together, these findings further support the capacity of retinal organoids to faithfully recapitulate key features of the human retina.

The use of retinal organoids to model Stargardt disease has been previously explored.^35^^,^^57^^,^^59^^,^^60^ In contrast to our results, Rui Chen and colleagues earlier described marked transcriptomic alterations in *ABCA4* patient-derived photoreceptors at DD300, identifying 2074 and 694 downregulated genes, as well as 501 and 92 upregulated genes, in cones and rods, respectively.^57^ Notably, a considerable fraction of these DEGs was not restricted to photoreceptors. However, the authors did not report DEGs in MGCs or astrocytes. One possible explanation for the discrepancies between their results and ours lies in the experimental design. Their scRNA-Seq analysis compared patient-derived organoids to non-isogenic healthy controls, whereas our study employed an isogenic system. While our organoids were processed at DD225, Rui Chen’s group examined organoids at approximately DD130 and DD300. Importantly, both the “early” and “late” cohorts included organoids differing in age by 15-58 days, a factor that may substantially influence retinal cell-type composition and maturation state. In addition, differences in differentiation protocols further complicate direct comparisons between studies.

Nevertheless, their GO analysis of photoreceptors revealed enrichment of apoptotic pathways and reduced neuronal functions, including processes related to development and microenvironment of glial cells, and intercellular communication in the organoids differentiated from patient hiPSCs carrying pathogenic variants in the *ABCA4* gene. These observations are consistent with our findings in non-photoreceptor retinal populations.

In fact, in our study MGCs exhibited 29 common DEGs, enriched in pathways related to glial microenvironment disruption, programmed cell death, and neuronal development, whereas astrocytes showed an even stronger response, with 66 shared DEGs across subclones, involved in similar functional categories and additionally highlighting cell–cell interaction pathways.

Among the dysregulated genes, *ADGRV1* was downregulated in both cell types. Beyond its established role in photoreceptor protein trafficking,^61^ ADGRV1 contributes to glutamate homeostasis in astrocytes, thereby supporting neuronal development.^62^ Its reduced expression suggests impaired neuron–glia communication and neuronal maturation, although the underlying mechanisms remain to be elucidated.

Another notable finding was the downregulation of *PCDHGA3,* encoding a protocadherin involved in cell adhesion, whose ubiquitination modulates cell–cell interaction.^63^  *PCDHGA3* expression was reduced across multiple retinal populations, including MGCs, astrocytes, amacrine cells, and the transient T2 cluster. In some of these populations, *PCDHGA3* expression was completely ablated, suggesting impaired retinal connections.

In addition to ferroptosis-related signatures, several apoptosis-associated GO terms were enriched in MGCs and astrocytes, potentially reflecting altered retinal development cell death. Consistently, *PHLDA1* was upregulated in both cell types, whereas *BNIP3* and *BNIP3L* were downregulated in MGCs and astrocytes, respectively, supporting cell-type-specific modulation of apoptotic signaling.

Taken together, our results show that the loss of ABCA4 protein from the photoreceptor disks is primarily associated with transcriptional changes in MGCs and astrocytes, rather than rods and cones. This might represent an early response to ABCA4 deficiency and/or subtle alterations in photoreceptor homeostasis, potentially reflecting a nonspecific reactive glial state (gliosis). This reactive response is characterized by altered expression of genes involved in neurodevelopmental processes and microenvironment of glial cells, highlighting the sensitivity of these cells to perturbations in the more resistant photoreceptors. These observations align with our recent findings in *USH2A*-13KO hiPSC-derived retinal organoids, in which loss of usherin isoform B from the connecting cilium resulted in only mild transcriptional alterations in rod photoreceptors, but a markedly stronger disruption of homeostasis in MGCs.^48^

The role of MGCs as early responders to photoreceptor stress and their capacity to exhibit gene dysregulation prior to photoreceptor dysfunction has been documented in inherited retinal diseases.^64^^,^^65^ For example, the *rd10* model of retinitis pigmentosa displayed early gliosis characterized by GFAP upregulation before the onset of extensive retinal degeneration, accompanied by reduced CREB phosphorylation and migration of MG cell bodies toward the ONL.^66^ Single-cell transcriptomic profiling in *rd10* retinas identified Müller glial transcriptional alterations during primary rod degeneration, including gliosis, immune dysregulation, and perturbations in iron metabolism.^67^

Consistent with these observations, scRNA-Seq analysis of *Abca4* mice models has shown that mutant MGCs display early transcriptional responses to light-induced retinal degeneration, including dysregulation of synaptic connections and extracellular matrix remodeling.^21^ In addition, mutations in the MGC-associated gene encoding CRALBP, a key component of the visual cycle, have been linked to autosomal recessive retinitis pigmentosa.^68^ Similarly, Royal College of Surgeons rats exhibited increased glutamine labeling within MGCs due to impaired glutamate degradation at stages preceding retinal degeneration.^69^ Together, these studies support the concept that Müller glia are highly sensitive to retinal stress and may undergo functional and transcriptional remodeling before detectable photoreceptor loss.

Although such early glial alterations have not been extensively characterized in the widely studied *Abca4* knockout mice, this may partly reflect the relatively mild and slowly progressive phenotype observed in these models, which can complicate the detection of subtle early-stage cellular responses. While we cannot exclude the possibility that some of the observed astrocytic and Müller glial changes are specific to the retinal organoid system, the available evidence from other inherited retinal degeneration models supports the interpretation that these alterations may represent genuine early disease-associated stress responses.

Our preliminary immunofluorescence analyses did not reveal statistically significant differences in GFAP or CD44 intensity, or in the percentage of cleaved caspase-3-positive cells between control and mutant organoids. However, the limited number of independent differentiations and organoids available for quantification likely reduced the statistical power to detect subtle alterations. Additional validation across multiple batches will be needed to determine whether the transcriptional changes identified by scRNA-Seq translate into protein-level and cellular phenotypes.

In vivo, STGD1 pathology depends on the transfer of photoreceptor-derived bisretinoids to the RPE through outer segment phagocytosis, leading to lysosomal dysfunction and oxidative stress in the RPE, and subsequent photoreceptor degeneration.^56^^,^^70^ Because retinal organoids lack a mature functional RPE, this pathogenic cascade is absent, which may explain the preserved transcriptional profiles of rods and cones despite loss of ABCA4 protein. The pronounced transcriptional alterations in MGCs and astrocytes may therefore represent an early, RPE-independent response that precedes photoreceptor death. Future co-culture models incorporating functional RPE will be important to determine whether ABCA4 deficiency ultimately triggers secondary photoreceptor degeneration.

Several limitations should be acknowledged. Although the use of isogenic controls reduces background genetic variability, only two independent *ABCA4* edited subclones were analyzed, leaving the possibility of clone-specific effects. Follow-up experiments with both additional independent mutant and unedited parental clones are essential to confirm the reproducibility of our findings, and specifically of the transcriptomic data. In fact, while immunofluorescence results were validated across 3 differentiations, scRNA-Seq was performed on a single batch of organoids. Differential expression analysis employed statistical testing at the level of individual cells, without accounting for the lack of full independence among cells originating from the same differentiation round, and therefore requiring caution in the interpretation of the results. Lastly, since expression of a truncated ABCA4 cannot be ruled out, the observed transcriptional alterations may reflect the loss of normal ABCA4 function, the presence of a truncated protein with altered biological properties, or a combination of both.

Nevertheless, retinal organoids offer a relevant system for evaluating novel gene therapies. To overcome the packaging limitations of large genes, such as *ABCA4,* dual AAV strategies have been developed, including intein-mediated protein trans-splicing, which enables full-length ABCA4 reconstitution,^35^ and has demonstrated both safety and therapeutic efficacy in preclinical models.^34^ Recent advances, such as mRNA trans-splicing^71^ and AAV with translocation linkage^72^, have further expanded the versatility and precision of large cargo delivery. In parallel, AAV-mediated HITI has successfully ameliorated the phenotype of mouse models of retinitis pigmentosa.^73^ Collectively, these studies highlight the therapeutic potential of gene therapy for inherited retinal disorders, while the *ABCA4* mutant retinal organoids here characterized provide a relevant human platform to test *ABCA4* HITI vectors.

## Conclusion

In conclusion, our data demonstrate that disruption of the *ABCA4* gene results in loss of ABCA4 protein from the photoreceptor outer segment disks, without inducing detectable transcriptional alterations in rods and cones. In contrast, MGCs and astrocytes exhibited significant transcriptomic changes, affecting neuronal development, intercellular communication, and programmed cell death. Further studies are needed to determine the functional consequences of these changes and their role in Stargardt disease.

## Supplementary Material

sxag045_Supplementary_Data

## Data Availability

The data underlying this article are available in the article and in its online supplementary material, except the scRNA-Seq data that are accessible at the GEO repository (GSE326273).
