# Supplementary material for: Loss of ABCA4 from photoreceptor discs is associated with glial transcriptomic changes in retinal organoids
Source: Stem Cells. 2026 Aug 11;44(9):sxag045. doi: 10.1093/stmcls/sxag045 (PMC13529370; doi:10.1093/stmcls/sxag045)
Supplement: Supplementary file 1 [file sxag045_supplementary_data.docx]

**Supplementary Materials**

**Loss of ABCA4 from photoreceptor discs is associated with glial transcriptomic changes in retinal organoids**

Rossella Valenzano ^1,5^, Andrew McDonald ^1,5^, Carmen Gallego ^1,5, †^, Charlotte A. Andriessen ^1^, Ioannis Moustakas ^2^, Aat A. Mulder ^3^, Harald M.M. Mikkers ^4^, Roman I. Koning ^3^, Hailiang Mei ^2^ and Jan Wijnholds ^1,^*

^1^ Department of Ophthalmology, Leiden University Medical Center, Albinusdreef 2, 2333 ZA Leiden, The Netherlands; r.valenzano@lumc.nl (R.V.); a.mcdonald@lumc.nl (A.M.); c.a.andriessen@lumc.nl (C.A.A.)

^2^ Department of Biomedical Data Sciences, Sequencing Analysis Support Core, Leiden University Medical Center, Albinusdreef 2, 2333 ZA Leiden, The Netherlands; i.moustakas@lumc.nl (I.M.);
h.mei@lumc.nl (H.M.)

^3^ Department of Cell and Chemical Biology, Electron Microscopy Facility, Leiden University Medical Center, Albinusdreef 2, 2333 ZA Leiden, The Netherlands; a.a.mulder@lumc.nl (A.A.M.); r.i.koning@lumc.nl (R.I.K.)

^4^ Department of Cell and Chemical Biology, Leiden University Medical Center, Albinusdreef 2, 2333 ZA Leiden, The Netherlands; h.mikkers@lumc.nl (H.M.M.M.)

^5^ Co-first author

* Correspondence: [j.wijnholds@lumc.nl](mailto:j.wijnholds@lumc.nl), Department of Ophthalmology, Leiden University Medical Center, Albinusdreef 2, 2333 ZA Leiden, The Netherlands

^†^ Present address: VectorY Therapeutics, Science Park 408, 1098 XH Amsterdam, The Netherlands

**Figure S1: Validation of *ABCA4-*mutant hiPSCs.** (A) Karyotyping analysis showing normal metaphases. (B) Copy number variations (CNV) analysis indicating no abnormalities in the mutant subclones. (C) Short tandem repeats showing derivation of the *ABCA4* lines from the ISO-CTRL.

**Alt text**: *ABCA4*-mutant hiPSCs show normal karyotypes, absence of detectable copy number variations, and confirmed genetic identity with the isogenic control line.

**Figure S2:** **Pluripotency, trilineage differentiation and off-target mutagenesis analyses of *ABCA4* hiPSCs subclones.** (A) Assessment of pluripotency markers Oct3-4, Nanog, and SSE4 by flow cytometry. (B) Trilineage differentiation of *ABCA4*-H10 and *ABCA4*-G3 subclones into mesoderm, endoderm and ectoderm. Scale bars: 50 µm. (C) Sanger sequencing of the genomic regions containing the top 5 off-target sites associated to the gRNA used in this study as predicted by the IDT “CRISPR-Cas9 guide RNA design checker” tool. Chromatograms indicate no indels around the expected cut site (dashed line in blue).

**Alt text**: Characterization of *ABCA4* hiPSC subclones showed expression of pluripotency markers, successful trilineage differentiation, and absence of indels at the predicted CRISPR off-target sites.

**Figure S3:** **Western Blot analysis of ABCA4 in control and *ABCA4*-mutant retinal organoids at DD240.** (A) Ponceau S staining of a polyvinylidene fluoride (PVDF) membrane following wet transfer of retinal organoid lysates. A total of 64 µg of protein lysate was loaded per lane for ISO-CTRL (n = 8), *ABCA4*-G3 (n = 8), and *ABCA4*-H10 (n = 2) organoids cultured until DD240. The staining pattern demonstrates efficient transfer of large proteins across all samples. (B) Detection of ABCA4 in lysates from ISO-CTRL, *ABCA4*-G3 and *ABCA4*-H10 organoids. No bands are visible at the expected molecular weight (MW) of 250 kD. (C) Detection of glyceraldehyde 3-phosphate dehydrogenase (GAPDH) in lysates from ISO-CTRL, *ABCA4*-G3 and *ABCA4*-H10 organoids. GAPDH bands are detected at the expected MW of 37 kD. (B, C) Arrowheads point at the expected band height for ABCA4 (B) and GAPDH (C). Detection was performed using the Clarity Western ECL Substrate.

**Alt text**: Efficient large protein transfer across control and *ABCA4*-mutant retinal organoid, but failed ABCA4 protein detection.

**Figure S4: Expression of *ABCA4*, *ROM1* and *PRPH2* in selected clusters of *ABCA4*-mutant and isogenic control organoids.** (A-C) Violin plots comparing the expression levels of *ABCA4* (A), *ROM1* (B), and *PRPH2* (C) in control and mutant rod photoreceptor cells. Significance was indicated as *p* < 0.01 (**), and ns (not significant). Adjusted p-values = 0,004 (**); all others = 1. (D-F) Violin plots comparing the expression levels of *ABCA4* (D), *ROM1* (E), and *PRPH2* (F) in control and mutant cone photoreceptor cells (all adjusted p-values = 1). (G-I) Violin plots comparing the expression levels of *ABCA4* in control and mutant Müller glial cells I (G), immature photoreceptors (H), and astrocytes (I) (all adjusted p-values = 1). Number of organoids used: ISO-CTRL *n* = 4; *ABCA4*-H10 *n* = 5; *ABCA4*-G3 *n* = 5 from 14 scRNA-Seq of the same differentiation round. Differential gene expression was evaluated at the single-cell level using Wilcoxon rank-sum test, with multiple-testing correction performed using the Benjamini–Hochberg method. Genes with an adjusted p-value < 0.05 were considered significantly differentially expressed.

**Alt text**: Violin plots compare *ABCA4*, *ROM1*, and *PRPH2* expression between control and *ABCA4*-mutant organoids in several clusters, detecting significant reduction of *ABCA4* transcript level only in the rods of subclone H10.

**Figure S5:** **Volcano plots showing differentially expressed genes in rods and cones of *ABCA4* subclones.** (A,B) Volcano plots indicating the downregulated (blue) and upregulated (red) genes in the rod photoreceptors of *ABCA4*-G3 (A) and *ABCA4*-H10 (B) compared to ISO-CTRL. (C,D) Volcano plots indicating the downregulated (blue) and upregulated (red) genes in the cone photoreceptors of *ABCA4*-G3 (C) and *ABCA4*-H10 (D) compared to ISO-CTRL. Statistical parameters: Wilcoxon rank-sum test, adjusted *p* value < 0.05, absolute log2FC threshold = 0.25. Number of organoids used: ISO-CTRL *n* = 4; *ABCA4*-H10 *n* = 5; *ABCA4*-G3 *n* = 5 from 14 scRNA-Seq of the same differentiation round.

**Alt text:** Volcano plots display differentially expressed genes in rod and cone photoreceptors of *ABCA4*-mutant subclones versus controls, highlighting upregulated and downregulated genes.

**Figure S6: Volcano plots showing differentially expressed genes in MGCs-I and astrocytes of *ABCA4* subclones.** (A, B) Volcano plots indicating the downregulated (blue) and upregulated (red) genes in the MGCs-I of *ABCA4*-G3 (A) and *ABCA4*-H10 (B) compared to ISO-CTRL. (C, D) Volcano plots indicating the downregulated (blue) and upregulated (red) genes in the astrocytes of *ABCA4*-G3 (C) and *ABCA4*-H10 (D) compared to ISO-CTRL. Statistical parameters: Wilcoxon rank-sum test, adjusted *p* value < 0.05, absolute log2FC threshold = 0.25. Number of organoids used: ISO-CTRL *n* = 4; *ABCA4*-H10 *n* = 5; *ABCA4*-G3 *n* = 5 from 14 scRNA-Seq of the same differentiation round. Genes with an adjusted p-value < 0.05 were considered significantly differentially expressed.

**Alt text:** Volcano plots display differentially expressed genes in Müller glial cells-I and astrocytes of *ABCA4*-mutant subclones versus controls, highlighting upregulated and downregulated genes.

**Figure S7: Pathway enrichment analysis of *ABCA4* MGCs-I.** (A,B) Bar plots showing the most significant pathway enrichment terms found in the MGCs-I cluster of *ABCA4*-H10 (A) and *ABCA4*-G3 (B). Similar terms have been grouped into categories labeled with colors. Number of organoids used: ISO-CTRL *n* = 4; *ABCA4*-H10 *n* = 5; *ABCA4*-G3 *n* = 5 from 14 scRNA-Seq of the same differentiation round.

**Alt text:** Bar plots show enrichment in terms grouped under microenvironment, programmed cell death, cell-cell interaction, neuron development, iron binding and transport, cell redox homeostasis, and synapses in the Müller glial cells-I of *ABCA4*-H10 organoids, and enrichment in terms belonging to microenvironment, respiratory chain, retinoid binding, programmed cell death, and synapses in the Müller glial cells-I of *ABCA4*-G3 organoids.

**Figure S8:** **Differential expression analysis of *ABCA4*-mutant MGCs-I compared to isogenic controls.**  Dot plots displaying DEGs associated with cell-cell interaction (A), neuron development (B), metabolic stress (C), retinoid binding (D), iron binding and transport (E), and programmed cell death (F). Dot plots (B) and (D) display additional DEGs retrieved from neuron development and programmed cell death terms that were not included in Figure 5, which only shows DEGs shared between subclones. The genes shown here represent subclone-specific DEGs. Dashed boxes highlight genes that did not exhibit differential expression in a specific subclone compared to ISO-CTRL. Number of organoids used: ISO-CTRL *n* = 4; *ABCA4*-H10 *n* = 5; *ABCA4*-G3 *n* = 5 from 14 scRNA-Seq of the same differentiation round. Differential gene expression was evaluated at the single-cell level using Wilcoxon rank-sum test, with multiple-testing correction performed using the Benjamini–Hochberg method. Genes with an adjusted p-value < 0.05 were considered significantly differentially expressed.

**Alt text**: Dot plots show subclone-specific differentially expressed genes in *ABCA4*-mutant Müller glial cells-I compared to controls, which were retrieved from terms belonging to cell-cell interaction, neuron development, metabolic stress, retinoid binding, iron binding and transport, and programmed cell death pathways.

**Figure S9: Pathway enrichment analysis of pooled *ABCA4*-mutant MGCs-I.** Bar plot showing the most significant pathway enrichment terms found in the MGCs-I cluster of pooled *ABCA4*-mutants compared to ISO-CTRL. Similar terms have been grouped into categories labeled with colors. Number of organoids used: ISO-CTRL *n* = 4; pooled *ABCA4*-mutant *n* = 10 from 14 scRNA-Seq of the same differentiation round.

**Alt text:** Bar plots show enrichment in terms grouped under microenvironment, neuron development, cell-cell interaction, iron binding and transport, and programmed cell death in the Müller glial cells-I of pooled *ABCA4*-mutant organoids.

**Figure S10: Differential expression analysis of pooled *ABCA4*-mutant MGCs-I compared to isogenic controls.**  Dot plots displaying DEGs associated with microenvironment (A), neuron development (B), cell-cell interaction (C), iron binding and transport (D), and programmed cell death (E). Due to the high number of genes, to ensure better clarity panel A shows only a selection of DEGs retrieved from microenvironment-related GO terms. Number of organoids used: ISO-CTRL *n* = 4; pooled *ABCA4*-mutant *n* = 10 from 14 scRNA-Seq of the same differentiation round. Differential gene expression was evaluated at the single-cell level using Wilcoxon rank-sum test, with multiple-testing correction performed using the Benjamini–Hochberg method. Genes with an adjusted p-value < 0.05 were considered significantly differentially expressed.

**Alt text:** Dot plots show differentially expressed genes in pooled *ABCA4*-mutant Müller glial cells-I versus controls, retrieved from terms belonging to microenvironment, neuron development, cell-cell interaction, iron binding and transport, and programmed cell death pathways.

**Figure S11: Pathway enrichment analysis of *ABCA4* astrocytes.** (A,B) Bar plots showing the most significant pathway enrichment terms found in the astrocytes of *ABCA4*-H10 (A) and *ABCA4*-G3 (B). Similar terms have been grouped into categories labeled with colors. Number of organoids used: ISO-CTRL *n* = 4; *ABCA4*-H10 *n* = 5; *ABCA4*-G3 *n* = 5 from 14 scRNA-Seq of the same differentiation round.

**Alt text:** Bar plots show enrichment in terms grouped under neuron development, cell-cell interaction, microenvironment, synapses, and programmed cell death in the Müller glial cells-I of *ABCA4*-H10 organoids, and enrichment in terms grouped under neuron development, cell-cell interaction, microenvironment, programmed cell death, glycolysis, and respiratory chain complex in the Müller glial cells-I of *ABCA4*-H10 organoids.

**Figure S12: Differential expression analysis of *ABCA4*-mutant astrocytes compared to isogenic controls.**  Dot plots displaying DEGs associated with neuron development (A), synapses (B), and glycolysis (C) that were not included in Figure 7, which only shows DEGs shared between subclones. The genes shown here represent subclone-specific DEGs. Dashed boxes highlight genes that did not exhibit differential expression in a specific subclone compared to ISO-CTRL. Number of organoids used: ISO-CTRL *n* = 4; *ABCA4*-H10 *n* = 5; *ABCA4*-G3 *n* = 5 from 14 scRNA-Seq of the same differentiation round. Differential gene expression was evaluated at the single-cell level using Wilcoxon rank-sum test, with multiple-testing correction performed using the Benjamini–Hochberg method. Genes with an adjusted p-value < 0.05 were considered significantly differentially expressed.

**Alt text**: Dot plots show subclone-specific differentially expressed genes in *ABCA4*-mutant astrocytes versus controls, highlighting pathways related to neuron development, synapses, and glycolysis.

**Figure S13: Pathway enrichment analysis of pooled *ABCA4*-mutant astrocytes.** Bar plot showing the most significant pathway enrichment terms found in the astrocytes cluster of pooled *ABCA4*-mutants compared to ISO-CTRL. Similar terms have been grouped into categories labeled with colors. Number of organoids used: ISO-CTRL *n* = 4; pooled *ABCA4*-mutant *n* = 10 from 14 scRNA-Seq of the same differentiation round.

**Alt text:** Bar plots show enrichment in terms grouped under neuron development, microenvironment, cell-cell interaction, and programmed cell death in the astrocytes of pooled *ABCA4-*mutant organoids.

**Figure S14: Differential expression analysis of pooled *ABCA4*-mutant astrocytes compared to isogenic controls.**  Dot plots displaying DEGs associated with neuron development (A), microenvironment (B), cell-cell interaction (C), and programmed cell death (E). Due to the high number of genes, to ensure better clarity panel A and B show only a selection of DEGs retrieved from neuron development- and microenvironment-related GO terms. Number of organoids used: ISO-CTRL *n* = 4; pooled *ABCA4*-mutant *n* = 10 from 14 scRNA-Seq of the same differentiation round. Differential gene expression was evaluated at the single-cell level using Wilcoxon rank-sum test, with multiple-testing correction performed using the Benjamini–Hochberg method. Genes with an adjusted p-value < 0.05 were considered significantly differentially expressed.

**Alt text**: Dot plots show differentially expressed genes in pooled *ABCA4*-mutant astrocytes versus controls, highlighting pathways involved in neuron development, microenvironment, cell-cell interaction, and programmed cell death.

**Figure S15: Assessment of gliosis and apoptosis in *ABCA4* retinal organoids at DD240.** (A) Representative immunofluorescence images of MGCs markers, GFAP (green) and CD44 (cyan) with and without DAPI (grey). Scale bars: 20 µm. (B) Quantitative analysis of CD44 fluorescence intensity normalized to the organoid area within the field of view. Number of organoids used: ISO-CTRL *n* = 10; *ABCA4*-G3 *n* = 5; *ABCA4*-H10 *n* = 3, from two rounds of differentiation. Group differences were assessed using the Kruskal-Wallis test followed by Dunn’s multiple comparisons test. Significance was indicated as ns (not significant). Statistical analysis: *p* = 0.2176, *p* = 0.2176, from left to right. (C) Quantitative analysis of GFAP fluorescence intensity normalized to the organoid area within the field of view. Number of organoids used: ISO-CTRL *n* = 10; *ABCA4*-G3 *n* = 5; *ABCA4*-H10 *n* = 3, from two rounds of differentiation. Group differences were assessed using the Kruskal-Wallis test followed by Dunn’s multiple comparisons test. Significance was indicated as ns (not significant). Statistical analysis: *p* = 0.8454, *p* = 0.8454, from left to right. (D) Representative immunofluorescence images of cleaved caspase-3 (green) with DAPI (grey). Regions outlined by dashed boxes are displayed at higher magnification in the corresponding zoomed panels below. Arrows in the bottom images indicate examples of cleaved caspase-3 staining appearing as puncta. Scale bars: top, 20 µm; bottom 6 µm. (E) Quantitative analysis of the percentage of cleaved caspase-3 positive cells in the outer nuclear layer (ONL, left), inner nuclear layer (INL, middle), and inner retina (right) of ISO-CTRL and *ABCA4* organoids. Number of organoids used: ISO-CTRL *n* = 10; *ABCA4*-G3 *n* = 6; *ABCA4*-H10 *n* = 3, from two rounds of differentiation. Group differences were assessed using the Kruskal-Wallis test followed by Dunn’s multiple comparisons test. Significance was indicated as ns (not significant). Statistical analysis: *p* > 0.999, for all comparisons. (B, C, E) Differentiations were treated as biological replicates and organoids derived from the same differentiation were treated as non-independent technical replicates. Organoid measurements are displayed as individual datapoints for transparency and were averaged within each differentiation prior to statistical analysis.

**Alt text:** Exploratory analysis shows no significant differences in GFAP, CD44, or cleaved caspase-3 levels between control and *ABCA4* retinal organoids, indicating unchanged gliosis and apoptosis at DD240.

**Table S1:** hiPSC line information.

| Line name | Description | Gender |
| --- | --- | --- |
| LUMC0004iCTRL10 = ISO-CTRL  (hPSCreg name: LUMCi029-B) | Control parental hiPSC line | Male |
| LUMC0004iCTRL10_*ABCA4^24^*^KO^ CLG3 = *ABCA4*-G3 | A 10-bp deletion–insertion introduces a premature stop codon in exon-24 of *ABCA4*.  c.3574-3583delinsTGA; p.(Val1192Ter) | Male |
| LUMC0004iCTRL10_*ABCA4^24^*^KO^ CLH10 = *ABCA4*-H10 | A 10-bp deletion–insertion introduces a premature stop codon in exon-24 of *ABCA4*.  c.3574-3583delinsTGA; p.(Val1192Ter) | Male |

Information on the hiPSC lines used in this study.

**Table S2:** Information related to the crRNA and donor template used to generate the *ABCA4*-mutant hiPSC lines and primers to assess the correct genomic mutation and absence of off-target mutagenesis.

| Name | Sequence (5’-3’) |
| --- | --- |
| *ABCA4* exon-24 crRNA | CTGGAGTTAGGTCATCGACG |
| *ABCA4* ssODN | AGTCATCCCATCCATCTGTTGCAGGGGACCTGCAGCTGCTCGTCTAAGGGTTTCTCCACCACGTGTCCAGCCCACTGATAACTCCAGAACAAGTCCTGGATGGTAAGGACTGGACGGGCCATACTTGGGTTCCGTCTGGCAGCCATCTCCCAG |
| *ABCA4* exon-24 Fw primer | GACCCTTGGAGGTTCTGCTC |
| *ABCA4* exon-24 Rv primer | TGCATCACCATGCTTCCACT |
| Off-target 1 (chr4:+98677999) Fw primer | AGGGTACAGGTGAGGGGAAA |
| Off-target 1 (chr4:+98677999) Rv primer | TCTTCATGTTGGTGAGCCCC |
| Off-target 2 (chr1:-118492932) Fw primer | ACTCCCTTTGTCAGCTCTACA |
| Off-target 2 (chr1:-118492932) Rv primer | ACACTAGGTATTGTTTAGTTGGTGT |
| Off-target 3 (chr4:+27953016) Fw primer | CAGCAGGTAACAGGCACTCA |
| Off-target 3 (chr4:+27953016) Rv primer | TTTCTTCATAGGTGAACAACTGTC |
| Off-target 4 (chr14:+83381666) Fw primer | CCATTGGAGGGAAAGATACACTAAG |
| Off-target 4 (chr14:+83381666) Rv primer | TCCAAGTGATTTTGTCTAGTATCATGA |
| Off-target 5 (chr7:-116836815) Fw primer | CCTGTTGGGAGAATGCTGGT |
| Off-target 5 (chr7:-116836815) Rv primer | TTTGATCCCCGAACCACACC |

Forward (Fw) and Reverse (Rv) primers used to confirm the CRISPR/Cas9-mediated insertion of the premature stop codon in *ABCA4* exon-24 and the absence of mutations in the top 5 predicted sites.

**Table S3:** List of materials used in this study.

| Materials | Source | Identifier |
| --- | --- | --- |
| tracrRNA | IDT | 1072532 |
| Nuclease-Free Duplex Buffer | IDT | 11-01-03-01 |
| SpCas9 Nuclease V3 | IDT | 1081058 |
| Neon™ Transfection System kit | Invitrogen | MPK10096 |
| Neon™ Transfection System | Invitrogen | MPK1025 |
| Matrigel hESC-Qualified Matrix | Corning | 354277 |
| mTeSR plus medium | STEMCELL Technologies | 100-0276 |
| Accumax | STEMCELL Technologies | 07921 |
| Gentle Cell Dissociation reagent | STEMCELL Technologies | 100-0485 |
| CloneR | STEMCELL Technologies | 05888 |
| Cryostor | STEMCELL Technologies | 07930 |
| DMEM/F12 | Life Technologies | 11320074 |
| DMEM(1X) + GlutaMAX | ThermoScientific | 10569010 |
| Fasudil HCl | Focus Biomolecules | 10-2137 |
| 40 µm cell strainer | pluriSelect | 43-10040-40 |
| Blebbistatin | abcam | ab120425 |
| Micro-molds | Merck | Z764000-6EA |
| MEM NEAA 100X | Life technologies | 11140-035 |
| Taurine | Merck | T0625 |
| Neurocult SM1 50X | STEMCELL Technologies | 05711 |
| N2 supplement 100X | Life technologies | 17502048 |
| Heparin | Merck | H-9399 |
| Smoothened agonist (SAG) | Selleck Chemicals | S7779 |
| Gamma secretase inhibitor IX (DAPT) | Selleck Chemicals | S2215 |
| Fetal Bovine Serum | Serana | S-FBS-CO-015 |
| Poloxamer 188 | Merck | P5556 |
| Retinoic acid | Merck | R-2625 |
| Antibiotic-antimycotic 100X | Merck | A5955 |
| Tissue-Tek O.C.T. Compound | Sakura Finetek | 4583 |
| Vectashield Antifade Mounting Medium | Vector Laboratories | H-1800-10 |
| Papain Dissociation kit | Worthington | I-LK 03150 |
| RIPA buffer | Merck | R0278 |
| cOmplete™, Mini, EDTA-free Protease Inhibitor Cocktail | Merck | 11836170001 |
| Clarity Western ECL Substrate | Bio-Rad | 1705060 |
| Clarity Max Western ECL Substrate | Bio-Rad | 1705062 |
| Pierce BCA Protein Assay kit | ThermoScientific | 23227 |
| Dithiothreitol (DTT) | Merck | 10197777001 |
| 4x Laemmli sample buffer | Bio-Rad | 1610747 |
| 4-20% Mini-PROTEAN TGX Precast Protein Gels | Bio-Rad | 4561094 |
| PVDF membrane | ThermoScientific | 10600023 |
| TWEEN 20 | Merck | P6585 |

**Table S4:** List of antibodies used in this study.

| Antibody | Dilution | Source | Identifier |
| --- | --- | --- | --- |
| Anti-CRALBP | 1:500 | Abcam | ab15051 |
| Anti-Recoverin | 1:600 | Millipore | AB5585 |
| Anti-MUPP1 | 1:150 | BD Transduction Laboratories | M98820 |
| Anti-Rhodopsin | 1:500 | Millipore | MAB5356 |
| Anti-R/G Opsin | 1:300 | Millipore | AB5405 |
| Anti-INPP5E | 1:200 | Proteintech | 17797-1-AP |
| Anti-ABCA4 (N-terminal) | 1:1000 | Millipore | MABN2440 |
| Anti-ROM1 | 1:300 | Proteintech | 21984-1-AP |
| Anti-PRPH2 | 1:400 | Proteintech | 18109-1-AP |
| Anti-GFAP | 1:400 | Dako | Z0334 |
| Anti-CD44 | 1:300 | ThermoScientific | MA4400 |
| Anti-cleaved caspase-3 | 1:250 | Cell Signaling | Asp175 |
| Anti-GAPDH | 1:2000 | Proteintech | 60004-1-lg |
| Goat anti-rabbit IgG (H+L) Highly Cross-Adsorbed Secondary Antibody, Alexa Fluor 488 | 1:1000 | Invitrogen | A-11034 |
| Goat anti-mouse IgG H&L, Alexa Fluor 555 | 1:1000 | Abcam | ab150118 |
| Anti-Oct3/4 | 1:25 | BD Biosciences | AB_2739320 |
| Anti-Nanog | 1:5 | BD Biosciences | AB_1645522 |
| Anti-SSEA4 | 1:25 | Miltenyi Biotec | AB_2811417 |
| Anti-Brachyury | 1:200 | Cell Signaling Technology | AB_2799983 |
| Anti-CDX2 | 1:500 | Cell Signaling Technology | AB_2797879 |
| Anti-Vimentin | 1:400 | Cell Signaling Technology | AB_10834530 |
| Anti-EOMES | 1:100 | Cell Signaling Technology | AB_2799974 |
| Anti-FOXA2 | 1:500 | Cell Signaling Technology | 10891055 |
| Anti-GATA-4 | 1:200 | Cell Signaling Technology | AB_2799108 |
| Anti-Nestin | 1:200 | Cell Signaling Technology | AB_2799037 |
| Anti-Pax6 | 1:200 | Cell Signaling Technology | AB_2797599 |
| Anti-mouse-IgGκ BP-HRP | 1:5000 | Santa Cruz Biotechnology | sc-516102 |

Product information about the antibodies used for flow cytometry, immunocytochemical, immunofluorescence, and Western Blot analyses.

**Supplementary Methods**

***1. Karyotyping analysis***

Normal karyotypes were confirmed by G-banding with 20 metaphases analyzed per cell line. Analysis was performed by the Section Genome Diagnostics, Clinical Genetics, LUMC, using the Leica Biosystems CytoVision (Leica Microsystems).

***2. Copy number variation analysis***

The genomic DNA of hiPSC lines was analyzed for copy number variations using the iCS-digital™ PSC 24-probes kit (Stemgenomics).

***3. Short Tandem Repeat (STR) analysis***

STR-analysis was performed by the LUMC Forensic Laboratory for DNA-research (FLDO), using the PowerPlex Fusion System 5C autosomal STR kit (Promega Corporation).

***4. Flow cytometry***

Single cells were prepared with Gentle Cell Dissociation Reagent (STEMCELL Technologies) and fixed and permeabilized using the FIX & PERM Cell Fixation & Permeabilization Kit (Invitrogen) following the manufacturer's instructions. Cells were stained with the directly conjugated antibodies against Oct3/4, SSEA4 and Nanog and subsequently measured using the MACSQuant VYB (Miltenyi Biotech). Data was analysed with FlowJo software.

***5. Trilineage differentiation***

To determine pluripotency, iPSC lines were differentiated into mesoderm, endoderm and ectoderm using the STEMdiff^TM^ Trilineage differentiation kit (STEMCELL Technologies) following the manufacturer’s instructions. Cells were fixed in PFA for 10 minutes and permeabilised with 0.01% Triton X-100. Cells were subsequently incubated with fluorophore-conjugated primary antibodies for 1 hr at RT. Nuclei were stained with VECTASHIELD® Antifade Mounting Medium with DAPI (Vector Laboratories). Images were captured by confocal microscopy (Leica SP8).

***6. Retinal Organoid Differentiation***

After three weeks of culture, hiPSCs were resuspended in mTeSR plus supplemented with 10 µM blebbistatin (Abcam). One million cells were seeded per agarose micro-mold (Merck) in a medium gradually transitioned to NIM1. Embryoid bodies (EBs) were transferred to Matrigel-coated 6-well plates in NIM1. From DD10, 100 nM of SAG (Selleck Chemicals) was added, and from DD16 cultures were maintained in NIM2 with SAG. Between DD21 and DD28, neuroepithelial structures were manually isolated and cultured in agarose-coated 48-well plates in NIM2. From DD35 to DD44, organoids were cultured in RLM1 + 1 µM retinoic acid (Merck), followed by RLM1 supplemented with 1 µM retinoic acid and 10 µM DAPT (Selleck Chemicals) until DD55. From DD56 to DD85, organoids were maintained in RLM1 with retinoic acid, after which cultures were switched to RLM2 with 0.5 µM retinoic acid. From DD120 onward, organoids were maintained in RLM2 until collection. Additional information on the Materials used can be found in Table S3.

***7. Transmission electron microscopy (TEM) analysis***

Samples were fixed at room temperature in 1.5% glutaraldehyde prepared in 0.1 M cacodylate buffer by adding an equal volume of double-strength fixative directly to the culture medium for 1 hour. Following fixation, samples were washed three times in 0.1 M cacodylate buffer and post-fixed for 1 hour on ice in 1% osmium tetroxide supplemented with 1.5% potassium ferricyanide in the same buffer. After three additional buffer washes, samples were dehydrated through graded ethanol solutions, followed by acetone and increasing concentrations of EPON resin (LX112), and finally infiltrated with 100% EPON. Organoids were embedded in molds filled with EPON and polymerized at 70 °C for 48 hours. Ultrathin sections (90 nm) were cut using a Reichert Ultracut S ultramicrotome (Leica Microsystems), contrasted with uranyl acetate and lead citrate, and imaged using a Tecnai T12 transmission electron microscope operated at 120 kV and equipped with a OneView camera (ThermoFisher Scientific). Serial images were acquired and stitched into composite micrographs and analyzed on ImageScope (v12.4.6).

***8. Western Blot analysis***

To obtain retinal organoid lysates with sufficient protein yield, pooled organoids were incubated in 150 µl (for n = 8) or 30 µl (for n = 2) of RIPA buffer on ice and homogenized using insulin needles combined with repeated mechanical pipetting for at least 30 minutes on a shaker. Subsequent Western blot procedures were performed as previously described [47]. 64 µg of retinal lysates were loaded onto the polyacrylamide (PAA) gel. Ponceau S staining was used to detect efficient wet transfer of large proteins from the gel to the polyvinylidene fluoride (PVDF) membrane. Primary antibodies were diluted in 5% milk in PBS-Tween 20 as follows: anti-ABCA4 (1:1000) and anti-GAPDH (1:2000). For protein detection, the Clarity Western ECL substrate was used prior to imaging with the Bio-Rad Chemidoc system. Specific signals for full-length or truncated ABCA4 were below detection level in the lysates from retinal organoids cultured under our experimental conditions.

***9. scRNA-Seq computational analysis***

Single cells were assigned to their sample of origin based on hashtag oligonucleotide (HTO) enrichment. Cells positive for a single HTO were classified as singlets and retained for further analysis. Quality control filtering was applied to keep only cells with nFeature_RNA between 500 and 8000, nCount_ RNA < 40000, and percent.mt <15. Data were normalized using the LogNormalize method with a scale factor of 30000, and the 2000 most variable genes were identified. Principal component analysis (PCA) was performed on scaled expression values, and the first 15 principal components were used for clustering. Nineteen transcriptionally distinct clusters were identified and visualized using UMAP. Cluster identities were assigned based on established retinal cell type marker genes identified using the FindAllMarkers function in Seurat. For downstream analyses, data were subset by cluster and experimental condition, and differential gene expression was assessed using Wilcoxon rank-sum test. Genes with an adjusted p-value below 0.05 were subjected to pathway enrichment analysis using gProfiler. In dot plots, dot size represents the proportion of cells expressing a given gene within each cluster, while color intensity reflects the average expression level.
